# Psychotherapy or medication for depression? Using individual symptom meta-analyses to derive a Symptom-Oriented Therapy (SOrT) metric for a personalised psychiatry

**DOI:** 10.1186/s12916-020-01623-9

**Published:** 2020-06-05

**Authors:** Nils Kappelmann, Martin Rein, Julia Fietz, Helen S. Mayberg, W. Edward Craighead, Boadie W. Dunlop, Charles B. Nemeroff, Martin Keller, Daniel N. Klein, Bruce A. Arnow, Nusrat Husain, Robin B. Jarrett, Jeffrey R. Vittengl, Marco Menchetti, Gordon Parker, Jacques P. Barber, Andre G. Bastos, Jack Dekker, Jaap Peen, Martin E. Keck, Johannes Kopf-Beck

**Affiliations:** 1 Max Planck Institute of Psychiatry, Kraepelinstraße 2-10, 80804 Munich, Germany; 2 International Max Planck Research School for Translational Psychiatry (IMPRS-TP), Munich, Germany; 3 Department of Neurology and Neurosurgery, Icahn School of Medicine at Mount Sinai, New York, NY USA; 4 Department of Psychiatry and Behavioral Sciences, Emory University School of Medicine, Atlanta, GA USA; 5 Department of Psychology, Emory University, Atlanta, GA USA; 6 Institute for Early Life Adversity Research, University of Texas Dell Medical School in Austin, Austin, TX USA; 7 Department of Psychiatry and Human Behavior, Brown University School of Medicine, Providence, RI USA; 8 Department of Psychology, Stony Brook University, Stony Brook, NY USA; 9 Department of Psychiatry and Behavioral Sciences, Stanford University, Stanford, CA 94304 USA; 10 Division of Psychology and Mental Health, The University of Manchester, Manchester, UK; 11 Department of Psychiatry, The University of Texas Southwestern Medical Center, Dallas, TX USA; 12 Department of Psychology, Truman State University, Kirksville, MO USA; 13 Department of Biomedical and Neuromotor Sciences, University of Bologna, Bologna, Italy; 14 School of Psychiatry, University of New South Wales, Sydney, NSW Australia; 15 Gordon F. Derner School of Psychology, Adelphi University, Garden City, New York, USA; 16 Contemporary Institute of Psychoanalysis and Transdisciplinarity of Porto Alegre, Porto Alegre, Brazil; 17 Department of Research, Arkin Mental Health Care, Amsterdam, Netherlands

**Keywords:** Major depressive disorder, Depression symptoms, Antidepressant medication, Psychotherapy, Systematic review, Meta-analysis, Precision psychiatry, Symptom-oriented therapy metric

## Abstract

**Background:**

Antidepressant medication (ADM) and psychotherapy are effective treatments for major depressive disorder (MDD). It is unclear, however, if treatments differ in their effectiveness at the symptom level and whether symptom information can be utilised to inform treatment allocation. The present study synthesises comparative effectiveness information from randomised controlled trials (RCTs) of ADM versus psychotherapy for MDD at the symptom level and develops and tests the Symptom-Oriented Therapy (SOrT) metric for precision treatment allocation.

**Methods:**

First, we conducted systematic review and meta-analyses of RCTs comparing ADM and psychotherapy at the individual symptom level. We searched PubMed Medline, PsycINFO, and the Cochrane Central Register of Controlled Trials databases, a database specific for psychotherapy RCTs, and looked for unpublished RCTs. Random-effects meta-analyses were applied on sum-scores and for individual symptoms for the Hamilton Rating Scale for Depression (HAM-D) and Beck Depression Inventory (BDI) measures.

Second, we computed the SOrT metric, which combines meta-analytic effect sizes with patients’ symptom profiles. The SOrT metric was evaluated using data from the Munich Antidepressant Response Signature (MARS) study (*n* = 407) and the Emory Predictors of Remission in Depression to Individual and Combined Treatments (PReDICT) study (*n* = 234).

**Results:**

The systematic review identified 38 RCTs for qualitative inclusion, 27 and 19 for quantitative inclusion at the sum-score level, and 9 and 4 for quantitative inclusion on individual symptom level for the HAM-D and BDI, respectively. Neither meta-analytic strategy revealed significant differences in the effectiveness of ADM and psychotherapy across the two depression measures. The SOrT metric did not show meaningful associations with other clinical variables in the MARS sample, and there was no indication of utility of the metric for better treatment allocation from PReDICT data.

**Conclusions:**

This registered report showed no differences of ADM and psychotherapy for the treatment of MDD at sum-score and symptom levels. Symptom-based metrics such as the proposed SOrT metric do not inform allocation to these treatments, but predictive value of symptom information requires further testing for other treatment comparisons.

## Background

“Major depressive disorder (MDD) is a debilitating disease” is one of the most frequent introductory phrases in psychiatric literature and rightfully so (e.g. [1–5]). Its lifetime prevalence varies across countries between 5.5 and 21% [6, 7], and it is estimated to be the second leading cause for years lived with disability [8], leaving no doubt about the importance and urgency of developing effective treatments. While research on potential new treatments such as esketamine and anti-inflammatory drugs is underway [9–11], antidepressant medication (ADM) and psychotherapy offer effective treatments for a majority of patients as shown in recent high-quality meta-analyses [12–14]. As evidence is mainly based on between-group comparisons (e.g. drug versus placebo), the specific patient-treatment match often remains unclear. Specifically, as patients react differently towards specific treatments in clinical practice, they often need to “cycle through” different types of ADM and/or psychotherapy to find the treatment that will eventually help them personally [15–17]. Consequently, there is an increasing awareness in psychiatric and psychotherapy research for the necessity of more personalised treatment approaches that align with Paul’s old but important question: “What works for whom?” [18].

Attempts have been made and are underway towards establishing such personalised psychiatric care. These cover a wide range of different approaches such as using big data sets and machine learning models to predict the MDD course from baseline self-reports [19, 20], explorative data-mining strategies in order to define decision trees for the treatment of depression [21], algorithm-based treatments associated with shorter treatment time [15], imaging-based functional connectivity indices for treatment selection [22], or statistical strategies to examine superiority between treatments depending on stratification variables [23, 24]. Among the latter is a promising attempt by DeRubeis and colleagues who developed the Personalised Advantage Index (PAI) by re-analysing data from a randomised controlled trial (RCT) of cognitive behavioural therapy (CBT) versus ADM [25]. The PAI constitutes the predicted difference in how patients would have benefitted from the treatment they received to the treatment they did not receive. In order to estimate the PAI for each patient across treatments, the authors used regression-based models with prognostic and prescriptive (treatment-moderating) variables as predictor variables and depression sum-scores at study endpoint (8 weeks) as outcome.

Four more recent studies on the treatment of depression have also estimated the PAI in RCTs of sertraline versus placebo [26], cognitive therapy versus interpersonal therapy [27], CBT versus psychodynamic therapy [28], and continuation-phase cognitive therapy versus fluoxetine for relapse prevention [29]. Throughout these studies, findings indicate that patients randomised to their optimal treatment (as suggested by the PAI) had clinically significantly better improvements in depression symptoms. Variables moderating treatment effects differed between studies and ranged from sociodemographic factors over life events up to personality and specific problems, symptoms, temperament, and comorbid conditions [25–29]. Beyond the PAI and analyses of single RCTs, individual patient data (IPD) meta-analysis work is currently underway by Weitz and colleagues who are trying to identify treatment moderators that indicate a combination of ADM and psychotherapy as more effective than monotherapy [30]. These studies thus describe strategies of using pre-treatment variables to inform treatment allocation. Nevertheless, the range of different, hardly overlapping variables between different studies stresses the sample dependency of results. It further reveals the necessity of replication in a prospective design to determine the clinical utility of stratified treatment allocation.

Besides the lack of prospective studies, we argue that previous studies potentially miss out on making use of the heterogeneity present within the construct of MDD. Specifically, all aforementioned studies using the PAI defined reductions in sum-scores on depression scales as the singular outcome variable to indicate treatment efficacy. Yet, individual depressive symptoms load differently on overall psychosocial impairment [31], so that clinical severity might not be best expressed by summing over individual depression scores [32]. This phenomenon gets aggravated when considering how low the content overlap in symptoms is between prominent depression scales [33]. Additionally, symptom combinations in MDD, for instance as defined by the Diagnostic and Statistical Manual of Mental Disorders 5th edition (DSM-5), allow two patients to present with a completely diverging symptom profile when considering the diametrically opposed symptoms such as insomnia and hypersomnia [34] within that compound symptom. This is not only of concern theoretically but a recent descriptive study in 3703 patients has estimated that of 1030 unique symptom combinations the most frequent symptom combination was only present in 1.8% of patients [35].

Utilising heterogeneity in symptom expression could offer additional, complementary insights for personalised treatment allocation of main treatments for depression. Empirically, two recent investigations have attempted to use information on symptom expression to differentiate effects of main treatments in re-analyses of RCTs comparing ADM and psychotherapy. In the first study, the authors compared treatment effects on distinct symptom clusters and results showed that cognitive therapy was better than both ADM and placebo in improving atypical vegetative symptoms (weight gain/increased appetite and hypersomnia) while there was no difference in clusters of (i) mood, (ii) cognitive/suicidal, (iii) anxiety, and (iv) typical vegetative symptoms (weight loss/decreased appetite and insomnia) [36]. These analysed symptom clusters still rely on assumptions of common factors underlying MDD, however, which has been criticised in recent research [37] and is suggestive of a move towards individual symptom analyses. Here, another re-analysis of individual symptom data indicated that ADM was better at reducing suicidality than CBT [38]. This latter study, however, was potentially underpowered for individual symptom analyses, and meta-analytic strategies could offer a more thorough approach. Nonetheless, these studies constitute promising attempts at delineating more symptom-specific treatment effects and are aided by other findings that show, for instance, that ADM efficacy as compared to placebo is most pronounced for the “depressed mood” symptom specifically [39].

In the current investigation, we wanted to develop and test what we term a *Symptom-Oriented Therapy* (SOrT) metric that aspires to quantify potential preference (or not) of ADM or psychotherapy similar to the PAI. Contrary to the PAI, however, the SOrT metric is not based on pre-treatment (moderating) variables but instead makes use of patients’ heterogeneity in symptom expression. Additionally, the SOrT metric is not based on re-analyses of individual RCT data but instead on effect size estimates obtained in meta-analyses across a number of RCTs to avoid dependency of results on individual study samples. In particular, we wanted to conduct a systematic review and meta-analysis with the aim of synthesising existing evidence from RCTs comparing ADM and psychotherapy in the treatment of depression. While we also aimed to update existing meta-analytic evidence of these treatments on depression sum-scores, a previously used strategy [40–42], our primary aim was to analyse treatment efficacy on individual depressive symptoms. As prior RCTs have largely measured depressive symptoms using the Hamilton Depression Rating Scale (here, HAM-D; also commonly abbreviated as HRSD or HDRS) and/or Beck’s Depression Inventory (BDI) [40], we focussed our investigation on these two scales.

Following this overview of symptom-based treatment differences and similarities, we then aimed to compute the meta-analysis-based SOrT metric to indicate preference (or not) for a specific treatment type (i.e. ADM or psychotherapy) at the individual-patient level. This was evaluated using both data from an existing depressed inpatient sample, the Munich Antidepressant Response Signature study [43], and data from a previous RCT of ADM versus psychotherapy, the Emory Predictors of Remission in Depression to Individual and Combined Treatments (PReDICT) study [44]. Here, the primary test of our metric was to compare patients allocated to optimal versus non-optimal treatment in the PReDICT study as defined using the SOrT metric. Our primary hypothesis was that patients receiving their optimal treatment had significantly better outcomes on depressive symptoms than those receiving non-optimal treatment. We hoped that developing the SOrT metric based on our results would be particularly useful for future researchers (and ultimately clinicians) to test and individualise treatments for patients suffering from MDD. In this way, our results could provide a more refined view on heterogeneity of MDD and hopefully move the field closer towards personalised medicine.

## Methods

### Step 1: Systematic review and meta-analysis of RCTs of ADM versus psychotherapy

#### Protocol registration

The protocol for this systematic review was registered on PROSPERO (identifier: CRD42019123905) [45].

#### Search strategy and study selection

We systematically searched PubMed Medline, PsycINFO, and Cochrane Central Register of Controlled Trials (CENTRAL) databases for randomised controlled trials (RCTs) of psychotherapy versus ADM in the treatment of depression. The search terms are presented in detail in Additional file 1: Table S1 in respective database grammar and align with the following search terms for PubMed Medline:

(depression[MeSH Terms] OR depressive disorder[MeSH Terms] OR mood disorder[MeSH Terms] OR affective* OR depress*) AND (psychotherapy [mh] OR (Cogniti* AND (technique* OR therap* OR restructur* OR challeng*)) AND (Antidepressive Agents [Pharmacological Action] OR agents, antidepressive[MeSH Terms] OR SSRI OR SNRI OR TCA OR “selective serotonin reuptake inhibitor” OR “selective norepinephrine inhibitor” OR “tricyclic antidepressant”) AND (randomized controlled trial[pt] OR controlled clinical trial[pt] OR randomized [tiab] OR randomly [tiab] OR trial [tiab] OR groups [tiab])

In addition to search of databases, we searched 352 RCTs mentioned on http://www.evidencebasedpsychotherapies.org/, a database created for psychotherapy RCTs and comparative trials [46]; hand-searched reference lists of retrieved RCTs and conference abstracts; searched for and, if necessary, contacted authors with published trial protocols; and wrote to prominent authors for unpublished data.

NK and JKB screened titles and abstracts of articles for the following inclusion and exclusion criteria: RCTs were required to (i) have at least one arm each for (individual and/or group) psychotherapy and ADM during part of the trial (e.g. crossover studies were allowed if data can be extracted before crossover), (ii) measure depressive symptoms using the Beck Depression Inventory (BDI) or Hamilton Depression Rating Scale (HAM-D), (iii) investigate adult depression, (iv) investigate major depression as a primary diagnosis without major medical comorbidity, (v) describe ethical approval and ascertainment of written informed consent, and (vi) include patients aged 18–75 years. For studies in languages other than English, we decided on a case-by-case basis whether we had the resources for translation of articles, which did not lead to further exclusions.

#### Risk of bias assessment

In order to assess the quality of RCTs and risk of bias, we evaluated included studies using the gold standard Cochrane Risk of Bias tool [47] while making specific adaptations of the tool to the context of psychotherapy research as was recently suggested by Munder and Barth [48]. The Cochrane Risk of Bias tool includes assessments of (i) random sequence generation, (ii) allocation concealment, (iii) blinding of participants and personnel, (iv) blinding of outcome assessment, (v) incomplete outcome data, (vi) selective reporting, and (vii) other biases. As blinding of participants and personnel is impossible in ADM versus psychotherapy comparisons, we assessed how differential expectations of patients and personnel about treatment were handled. Here, we focussed on bias being introduced by way of the study design, because wait-list control and combined treatment (i.e. ADM *and* psychotherapy) might bias participants towards expecting less or more treatment effects than single treatments (ADM *or* psychotherapy), respectively. Additionally, we assessed “bias due to deviations from intended interventions” (e.g. changes in treatment adherence or integrity), which has recently been added to the Cochrane Risk of Bias tool 2.0 and is particularly relevant for complex interventions like psychotherapy [48, 49]. First, NK and JKB independently assessed included studies for their risk of bias. In a second step, assessments were compared and inconsistencies discussed and resolved.

#### Data acquisition and extraction

The main outcome for systematic review and meta-analyses were individual *symptom* data. Of note, individual *symptom* data—as required for our analyses—are not equal to individual *patient* data (IPD). While IPD have one row per participant, the format we required for our analyses is similar to frequency tables per symptom per treatment group; that is, one row per symptom, one column per symptom severity increment (e.g. 0, 1, 2, and 3 for all 21 BDI items) per treatment group, and cells indicating the number of patients indicating this symptom severity at end of treatment.

As depression outcome is usually not reported on the described individual *symptom* level, which was, however, required for our analyses, we only expected to be able to obtain a subset of data through extraction from manuscripts. The extracted variables from manuscripts were (i) study design and population, (ii) type and dosage of psychotherapy and ADM, (iii) study duration and follow-up, (iv) depression questionnaires used, (v) sample sizes of treatment arms at baseline and study endpoint, (vi) means and standard deviations of depression (sum-scores) at least at baseline and study endpoint (BDI and/or HAM-D), (vii) comorbidities (axis I and II disorders), (viii) age and sex of study participants, (ix) inclusion and exclusion criteria, (x) handling of missing data, dropouts, and use of intention-to-treat analysis, and (xi) researcher allegiance (i.e. main investigators’ training). NK and JKB independently extracted these data and subsequently discussed and resolved inconsistencies. If relevant data were only present in graph format and we did not get a response from authors, data were extracted using a reliable software tool [50].

Contacting authors for data (esp. individual *symptom* data) was also done in a standardised manner to maximise available data for this investigation (Additional file 2).

#### Statistical analysis

The effect size for meta-analysis of depression is usually the standardised mean difference (SMD) when using sum-scores of depression scales. Although we wanted to provide an updated quantitative synthesis of depression sum-scores in the comparison of psychotherapy and pharmacotherapy using SMDs, our primary aim was to evaluate treatment differences at the individual symptom level. The SMD, however, does not address the potential non-normality of individual symptom items, which are on an ordinal but not necessarily interval scale. To that end, we have chosen to calculate proportional odds ratios (pORs) as effect sizes of first choice; these are appropriate for ordinal data if the proportionality assumption is met (i.e. if steps in symptom severity are similar) [51, 52]. pORs might be thought of as average ORs of all possible item severity steps (e.g. from 0 to 1, from 1 to 2, etc.).

In addition to pORs, we also presented analyses based on SMDs and "normal" ORs (after median split of symptom items) as sensitivity analysis. While SMDs and normal ORs are not ideal for this type of meta-analysis due to potentially violated assumptions and loss of power, respectively, they are more commonly used than pORs, so might offer additional insights to readers and highlight potential method dependency and robustness of the results. To allow comparability between metrics, we converted between (p)ORs and SMDs (referred to in results as converted (p)ORs and converted SMDs) according to the formula SMD = ln(OR)/1.81 [53]. As BDI and HAM-D have partially overlapping symptoms, we also performed a “spill over” sensitivity analysis by comparing individual symptom effect sizes between these scales for all comparable items. For instance, meta-analyses of “feelings of guilt” items in BDI and HAM-D should show comparable effect sizes towards ADM, psychotherapy, or neither. Additional file 3: Tables S2-S3 highlight scale differences and similarities on the item level, which authors NK and JKB have evaluated with reference to a prior content analysis of depression scales [33]. In addition to BDI and HAM-D comparability, we also expected differences in the BDI, depending on whether BDI-I or BDI-II was used. Comparable items of BDI-I and BDI-II were aggregated if possible but separated if necessary (Additional file 3: Table S5).

Meta-analyses were conducted using the *metafor* package [54] in *R* [55]. For meta-analyses, effects were weighted based on study sample size (i.e. inverse variance method) and we used a random-effects approach. Of note, we only analysed available data at the study endpoint (i.e. completer data) as intention-to-treat approaches for individual symptom items would have made analyses too complex and we were not aware of any specific procedures for individual symptom items. We hoped, however, that missing data at study endpoint (e.g. through dropout) was less of a problem for our comparison of two active treatments, which should have had relatively more similar levels of dropout as compared to, for instance, studies with wait-list control arm.

We investigated heterogeneity between studies using Cochrane’s *Q* and the *I*^*2*^ statistic. Publication bias was tested for meta-analysis of the sum-score depression outcome by visual funnel plot inspection and using Egger’s test [56].

### Step 2: Development and validation of the Symptom-Oriented Therapy (SOrT) metric

#### Computation of the SOrT metric

If our meta-analyses of individual depressive symptoms demonstrated treatment differences between ADM and psychotherapy, this would have potential benefit for the development of individualised treatment. To that end, we wished to provide researchers with a meta-analysis-based tool to compute what we term *Symptom-Oriented Therapy* (SOrT) metric. This metric is based on the quantitative results from meta-analyses, which served as weightings for patients’ individual depressive symptoms (on either HAM-D or BDI). Specifically, computation of the SOrT metric followed the formula *SOrT* = ∑_*i*_*m*_*i*_*s*_*i*_, where *m* equals the meta-analytic effect size (favouring ADM or psychotherapy) as (converted) SMD, *s* equals the symptom score on BDI or HAM-D, and *i* equals a specific symptom item. Of note, *m* is defined as converted SMD and not pOR because SMDs follow a linear scale centred at zero, which allows more feasible computation and interpretation. See Additional file 4 for a detailed rationale and discussion of the SOrT metric, potential extensions of the formula, and hypothetical computation examples (Additional file 4: Table S5).

#### Validation in MARS study

We computed SOrT scores for patients who took part in the MARS study, a subset of which has data available on both the BDI and HAM-D measures and was targeted in this validation step (*n* = 407). The MARS project was a naturalistic inpatient study of MDD patients conducted between years 2000 and 2015 at the Max Planck Institute of Psychiatry (Munich, Germany), the Bezirkskrankenhaus Augsburg (Germany), and the Klinikum Ingolstadt (Germany) [43]. Participating patients received various psychological assessments, and genetic and neuroendocrine measures were obtained with the goal of identifying (i) drug response predictors and (ii) subgroups of patients with similar biological pathophysiology.

We (i) provided descriptive statistics of patients’ SOrT scores for BDI and HAM-D based on the MARS psychiatric inpatient sample, (ii) compared these on important clinical and demographic characteristics, and, most importantly, (iii) cross-validated the SOrT metric for BDI and HAM-D. This cross-validation was performed by computing (i) correlations between SoRT scores of scales and (ii) differences of BDI and HAM-D SOrT scores on an individual person level, respectively. Based on this, we were able to determine potential scale dependency or independency of the SOrT metric, which, in turn, provided an indication of its value as a tool for further research.

#### Validation in PReDICT study

We computed SOrT scores for patients who took part and were randomised in the PReDICT study (*n* = 344) and performed analyses on all patients who completed the RCT (*n* = 234). PReDICT was an RCT comparing CBT versus escitalopram versus duloxetine for the treatment of depression [44, 57]. For validation and in line with the setup of this investigation, ADM groups of escitalopram and duloxetine were pooled. SOrT score computation was done using meta-analytic effect estimates based on all retrieved studies *except* the PReDICT study, so that validation of the clinical utility of our metric was done independently from the meta-analytic “training” samples. Similar to the approach for the PAI used by DeRubeis et al. [25], we divided patients into those who received their optimal treatment (i.e. the SOrT score valence matches the treatment group) versus those who received their non-optimal treatment (i.e. the SOrT score valence does not match the treatment group). Doing this allowed us to compare outcome at end of treatment as suggested by HAM-D sum-scores of the optimal versus non-optimal groups and test for significant differences using a simple independent samples *t* test. In a second step, we followed the same procedure with a more restrictive subsample that was more extreme in their SOrT scores. Specifically, we used the two-thirds of patients with more extreme SOrT scores and compared optimal versus non-optimal treatment allocation groups. These procedures were applied both for SOrT scores created from BDI and for SOrT scores created from HAM-D. In sum, this allowed us to see whether the SOrT metric might pose a clinically significant benefit for treatment allocation.

### Timeline (steps 1 and 2)

We describe the planned timeline for conductance of this registered report as well as timeline adherence in Additional file 5.

## Results

### Step 1: Systematic review and meta-analysis of RCTs of ADM versus psychotherapy

#### Literature search

**Fig. 1:**
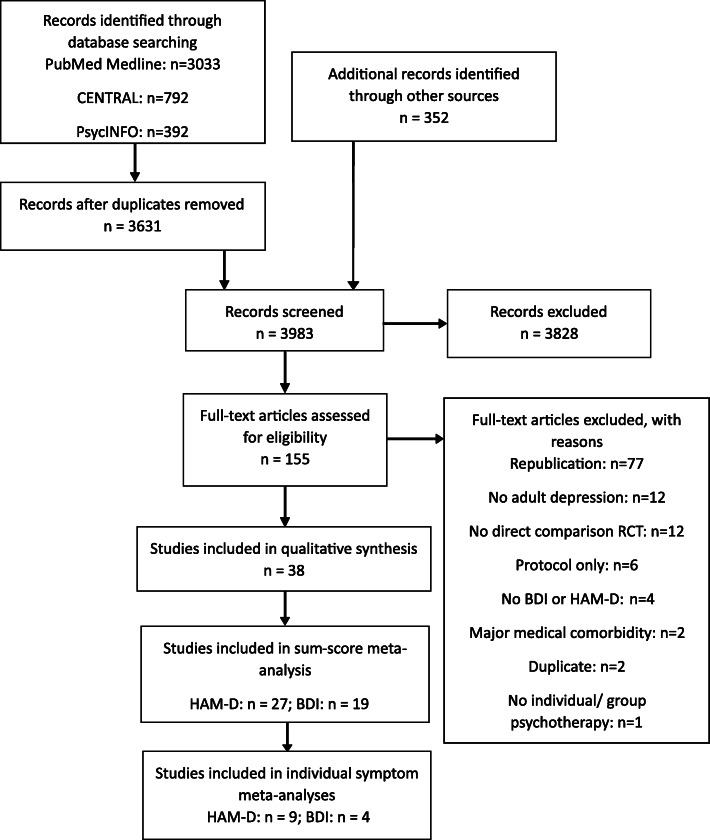
Adapted PRISMA flow diagram

The literature search was conducted on 31 January 2019 and revealed 4567 reports in total. Following duplicate removal, screening, and full-text assessments, we included 38 studies in our qualitative synthesis [57–94]. For our quantitative synthesis on the sum-score level, this was reduced to 27 studies with information on the HAM-D and 19 studies with information on the BDI. After corresponding with original authors to obtain individual symptom-level data, we were able to use 9 and 4 studies with HAM-D and BDI information, respectively, for our symptom-level meta-analyses. Of note, studies that reported data on both HAM-D and BDI were included in sum-score and symptom-specific analyses of both scales. Figure 1 shows the PRISMA flow diagram, and Additional file 6: Table S6 provides details of the included studies.

### Risk of bias

**Fig. 2:**
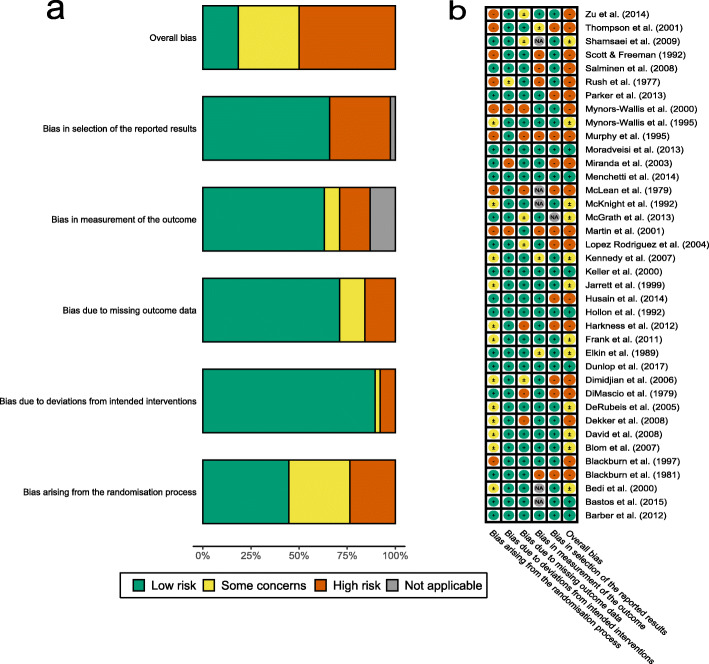
Risk of bias ratings **a** overall and **b** for specific studies

Results from Cochrane Risk of Bias tool ratings by NK and JKB are presented in Fig. 2 for individual risk of bias categories. Most studies have high overall risk of bias or reason for some concerns. Considering that the overall risk of bias takes forward ratings of individual bias categories, however, it is unsurprising that individual bias categories are more diverse. In particular, low risk of bias arises from deviations from intended interventions while selection of reported results and randomisation process ambiguities were more concerning.

### Sum-score meta-analyses

**Fig. 3:**
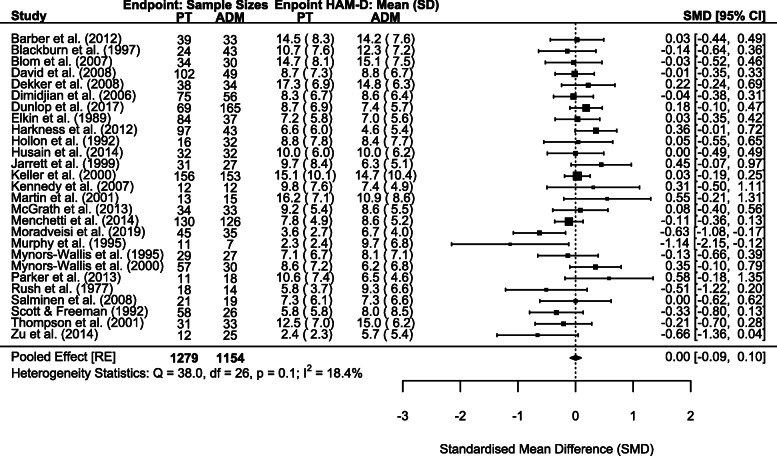
Forest plot of HAM-D sum-score meta-analysis

**Fig. 4:**
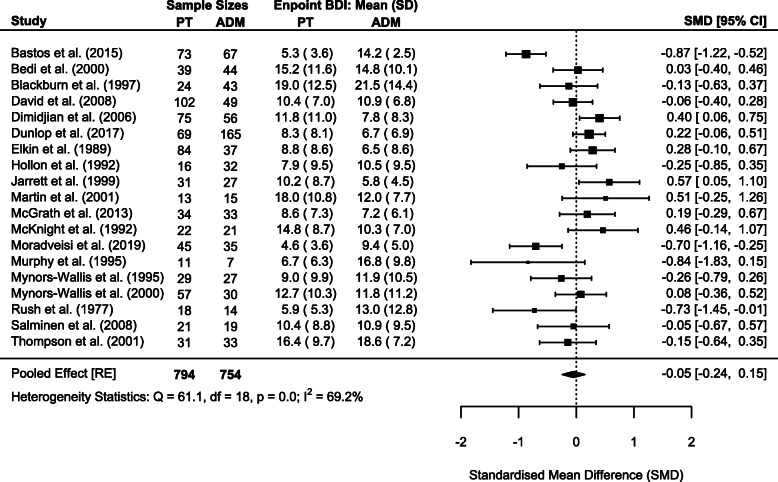
Forest plot of BDI sum-score meta-analysis

As previously done, we repeated sum-score meta-analyses for both HAM-D and BDI scales whenever outcome data were available in original studies. This allowed meta-analysis for 27 and 19 studies with 2433 and 1548 patients for HAM-D and BDI, respectively. Corresponding to completer only analyses on the individual symptom level, endpoint statistics for completers only were used in meta-analyses. We deviated from this procedure for 1 study, which only reported intention-to-treat statistics at endpoint [86]. Meta-analyses did not reveal significant differences in endpoint depressive symptom severity between psychotherapy and ADM for the HAM-D (SMD = 0.00, 95% CI − 0.09–0.10; Cochrane’s *Q* = 38.0, degrees of freedom [df] = 26, *p* = 0.060; *I*^2^ = 18.4%; Fig. 3) and the BDI (SMD = − 0.05, 95% CI − 0.24–0.15; Cochrane’s *Q* = 61.1, df = 18, *p* < 0.001; *I*^2^ = 69.2%; Fig. 4).

We assessed publication bias for HAM-D and BDI sum-score meta-analyses by visual funnel plot inspection (Additional file 7: Fig. S1-S2) and Egger’s test; neither revealed indications of publication bias in favour of psychotherapy or ADM (HAM-D: *z* = − 0.81, *p* = 0.418; BDI: *z* = − 0.72, *p* = 0.474).

Beyond these pre-registered analyses, we further explored sum-score meta-analysis results by evaluating differential dropout between study arms. In particular, we performed meta-regression analyses including percentage of greater dropout in ADM compared to psychotherapy as moderator. These results revealed significant moderation of differential dropout for the HAM-D (*p* = 0.018) but not for the BDI (*p* = 0.124; Additional file 7: Table S7). Importantly, RCTs with greater dropout in their ADM arm(s) were those with more favourable effects for psychotherapy and vice versa. Additional file 7: Figs. S3-S4 visualise results for sum-score meta-analyses for HAM-D and BDI, respectively. We also explored differential dropout in a separate meta-analysis of 35 studies with information on baseline and endpoint sample sizes. Overall, 458 of 2163 (21.2%) and 557 of 2133 (26.1%) patients dropped out from psychotherapy and ADM arms, respectively. This difference was significant and indicated that psychotherapy was more acceptable to patients (OR = 0.74, 95% CI 0.59–0.92; Cochrane’s *Q* = 57.7, df = 34, *p* = 0.007; *I*^2^ = 40.8%; see Additional file 7: Fig. S5 for the forest plot).

### Individual symptom meta-analyses

**Fig. 5:**
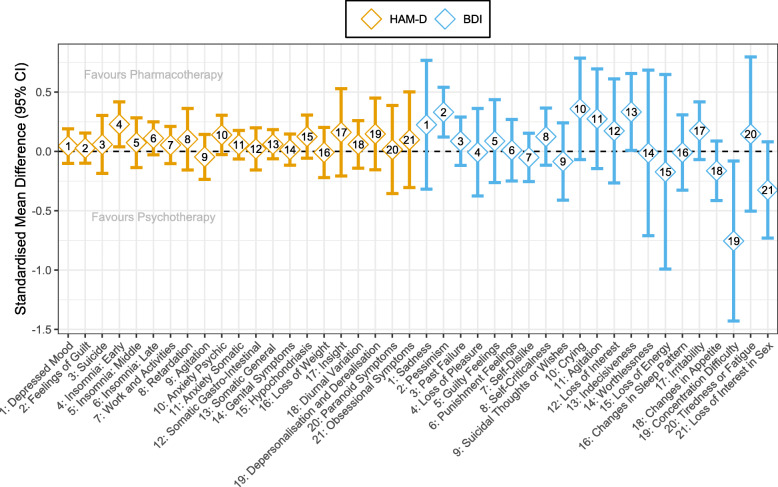
Pooled effect sizes (pORs converted to SMDs) on individual symptom level

We conducted individual symptom meta-analyses for 9 and 4 studies providing individual symptom data for HAM-D and BDI, respectively. Importantly, the number of studies and, correspondingly, the number of patients included in meta-analyses varied across symptoms with ranges of 421–1166 (median = 1166) and 379–502 (median = 501) completer patients for HAM-D and BDI items, respectively. The reasons for differing sample sizes across symptoms varied for the following reasons: We had data from fewer studies assessing the BDI, questionnaire versions differed, and the lack of variance in a particular symptom of a particular study necessitated the removal of such a study from a meta-analysis. Regarding differing questionnaire versions, for instance, some studies used a HAM-D version with more than 17 items. For the BDI, the items differ in versions I and II. Aggregated pooled meta-analytic effect sizes can be seen in Fig. 5. Additional file 8: Tables S8-S9 include forest plots with sample sizes and heterogeneity statistics for each symptom of HAM-D and BDI, respectively. Additional file 8: Fig. S6 shows effect size comparisons of HAM-D and BDI symptoms per symptom category (symptom categories as defined in Additional file 3: Table S2). These comparisons indicated similar effect sizes between symptom categories of guilt, loss of energy, and suicidality while there was a strong divergence between symptom categories of work and interests and loss of libido.

As meta-analytic results indicate, few symptoms showed nominally significant differences between psychotherapy and ADM (favouring ADM: HAM-D item 4 [insomnia: early], BDI item 2 [pessimism] and BDI item 13 [indecisiveness]; favouring psychotherapy: BDI item 19 [concentration difficulty]). Importantly, after family-wise error correction (using the Benjamini-Hochberg method) [95], none of these differences remained significant (all *p* > 0.05).

### Sensitivity analyses

To determine the influence of choice of effect size metric, we repeated individual symptom meta-analyses using “simple” ORs and SMDs in comparison to our metric of choice, the proportional OR (pOR). These sensitivity analyses provided a technical replication of our results indicating that choice of pOR as effect size metric was not driving our results (see Additional file 8 for details).

### Exploratory comparison with Boschloo et al. [96]

Following pre-acceptance of this registered report, Boschloo et al. published a highly similar report to this study as RCTs comparing CBT (as one form of psychotherapy) versus ADM were meta-analysed on the individual symptom level [96]. The HAM-D was used as the sole outcome rating scale, but SMDs between treatments were calculated as differences in symptom scores from baseline to study endpoint.

As we deemed it important for the field, we performed an exploratory comparison of our results with theirs. Details of this exploratory comparison are provided in Additional file 8. In sum, symptom-specific effect sizes from our meta-analyses were not associated with effect sizes from Boschloo et al. and only with small-to-moderate correlation when we restricted our meta-analysis to RCTs investigating CBT only.

### Step 2: Development and validation of the Symptom-Oriented Therapy (SOrT) metric

#### Validation in MARS study

We validated the SOrT metric using (i) a descriptive summary, (ii) assessment of clinical and demographic associations with our metric, and (iii) cross-validation between BDI- and HAM-D-based SOrT scores.

**Fig. 6:**
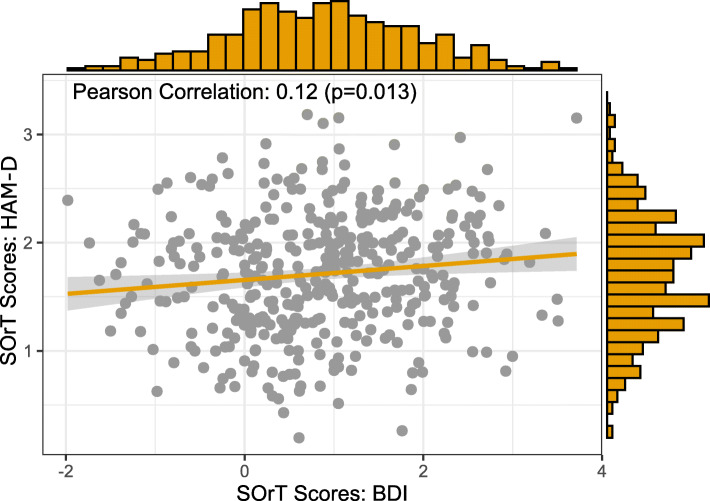
Association and distributions of HAM-D (*y*-axis) and BDI (*x*-axis) SOrT scores in the MARS sample

First, HAM-D and BDI SOrT metrics were approximately normally distributed in the sample (HAM-D: min = 0.20, median = 1.72, mean = 1.71, max = 3.18, SD = 0.55; BDI: min = − 1.98, median = 0.87, mean = 0.86, max = 3.72, SD = 1.04; Fig. 6). Contrary to our expectations, however, SOrT metric distributions were not centred around 0 and, in case of HAM-D SOrT scores, did not even include 0. A likely reason for the distributions not being centred around 0 is that most symptom-specific effect sizes were positive (i.e. favouring ADM) with 15/17 positive effect sizes for the HAM-D and 12/21 for the BDI. Second, we assessed clinical and sociodemographic correlates of respective SOrT scores. Importantly, we found high and small-to-moderate correlations between SOrT scores to their respective scale (HAM-D: Pearson’s *r* = 0.81, *p* < 0.001; BDI: Pearson’s *r* = 0.35, *p* < 0.001), which likely arises as an artefact of most effect sizes being positive. This also counters our initial goal of creating a treatment allocation metric (indicating psychotherapy versus ADM) as opposed to a mere measure of symptom severity. To highlight the distinction between a treatment allocation metric versus symptom severity associations, we report linear regression analyses, unadjusted and adjusted for baseline HAM-D and BDI sum-scores, of SOrT metric outcome on different sociodemographic and clinical predictor variables (Table 1). These analyses failed to reveal any consistent associations with our metric. Third, the small correlation of HAM-D and BDI SOrT scores (Pearson’s *r* = 0.12; cf. Fig. 6) was below the level considered meaningful for two treatment allocation metrics with identical goals (i.e. indicating favourable treatment by psychotherapy or ADM). In sum, our SOrT metric did not seem to be a valid measure with any clinical or sociodemographic correlates.

**Table 1:** Linear regression analyses of SOrT scores on sociodemographic and clinical predictor variables in the MARS sample

	HAM-D SOrT metric outcome				BDI SOrT metric outcome			
	Unadjusted		Adjusted^†^		Unadjusted		Adjusted^†^	
Predictor variable	Beta (SE)	*p*	Beta (SE)	*p*	Beta (SE)	*p*	Beta (SE)	*p*
Sociodemographic								
Female sex	0.125 (0.054)	0.021	0.044 (0.032)	0.167	− 0.084 (0.103)	0.414	− 0.173 (0.097)	0.075
Age	> 0.001 (0.002)	0.983	− 0.001 (0.001)	0.631	0.007 (0.004)	0.074	0.008 (0.003)	0.027
Married	0.025 (0.055)	0.648	− 0.045 (0.033)	0.176	− 0.103 (0.105)	0.328	−0.035 (0.099)	0.725
Education: German A-levels	−0.136 (0.055)	0.014	− 0.025 (0.034)	0.447	0.035 (0.106)	0.742	0.109 (0.100)	0.276
Clinical								
Baseline HAM-D sum-score	0.075 (0.003)	< 0.001	–	–	0.021 (0.009)	0.017	− 0.002 (0.009)	0.815
Baseline BDI sum-score	0.019 (0.002)	< 0.001	0.004 (0.002)	0.022	0.036 (0.005)	< 0.001	–	–
Discharge HAM-D sum-score	0.011 (0.005)	0.026	0.002 (0.003)	0.590	0.014 (0.010)	0.152	0.006 (0.009)	0.560
Discharge BDI sum-score	0.006 (0.003)	0.087	0.002 (0.002)	0.443	0.011 (0.007)	0.087	− 0.003 (0.007)	0.620
History of depression	− 0.011 (0.069)	0.869	0.049 (0.039)	0.214	0.082 (0.130)	0.526	− 0.069 (0.123)	0.576
Weeks of hospitalisation	0.010 (0.004)	0.009	< 0.001 (0.002)	0.829	0.010 (0.007)	0.173	> − 0.001 (0.007)	0.891
Anxiety (HAMA)	0.030 (0.003)	< 0.001	− 0.003 (0.003)	0.325	0.010 (0.006)	0.112	− 0.001 (0.006)	0.817

Separate linear regression analyses were performed for each predictor variable. Model intercepts are not reported to simplify result interpretation. Binary predictor variables are termed in a way that makes dummy coding obvious (e.g. female sex: 0 = male, 1 = female). *Abbreviation*: HAMA Hamilton Anxiety Rating Scale. ^†^Models were adjusted for respective (HAM-D or BDI) baseline symptom covariate

#### Validation in PReDICT study

The SOrT metric was further evaluated in data from the PReDICT trial [44, 57]. Again, SOrT scores were computed based on patients’ baseline symptom scores on HAM-D and BDI. Importantly, however, SOrT scores were based on effect sizes of individual symptom meta-analyses excluding PReDICT data to ascertain independence of our validation data (see comparison of effect sizes in Additional file 9: Table S11). As with the MARS sample, distributions and correlations of HAM-D and BDI SOrT scores are visualised in Additional file 9: Fig. S11. Contrary to our findings in MARS, we found a small, negative correlation between HAM-D and BDI SOrT scores (Pearson’s *r* = − 0.13, *p* = 0.054). Distributions were comparable for the HAM-D-based SOrT metric (min = 0.13, median = 1.15, mean = 1.15, max = 2.19, SD = 0.34) and more negative and variable for the BDI-based SOrT metric; this is consistent with a smaller number of trials included in BDI meta-analyses (min = − 5.27, median = − 0.98, mean = − 1.04, max = 2.09, SD = 1.39).

To compare optimal versus non-optimal SOrT-based treatment allocation, we initially pre-registered a sample split based on SOrT score valence (i.e. positive scores indicating optimal allocation to ADM and vice versa). As SOrT score distributions highlight, however, this approach was only possible for the BDI (23% of patients with positive SOrT scores) but not for the HAM-D (0% of patients with positive SOrT scores). Consequently, we decided to report an additional, exploratory classification of “optimal” versus “non-optimal” treatment allocation based on a SOrT score median split (i.e. patients above the median optimally treated by ADM and vice versa). Pre-registered and exploratory comparisons are reported in Table 2. None of these analyses revealed any potential benefit of allocating patients to treatment based on their SOrT scores. We performed additional linear regression analyses of these comparisons, unadjusted and adjusted for the HAM-D at baseline, to delineate potential dependency of the SOrT metric on symptom severity (Additional file 9: Tables S12-S14). These results align with pre-registered *t* tests.

**Table 2:** Evaluation of SOrT-based treatment allocation

		12-week HAM-D, mean (SD)		
Method	Pre-registered	Optimal	Non-Optimal	*p* value^†^
HAM-D-based SOrT allocation				
Valence	Yes	–	–	–
Median split	No	8.50 (6.41)	7.17 (5.86)	0.091
Extreme groups	Yes	8.86 (6.65)	7.08 (5.85)	0.074
BDI-based SOrT allocation				
Valence	Yes	7.62 (6.55)	8.03 (5.97)	0.617
Median split	No	7.63 (6.14)	8.09 (6.24)	0.563
Extreme groups	Yes	7.01 (6.16)	7.66 (6.15)	0.510

^†^*p* values are based on independent samples *t* tests

### Exploratory analyses

We conducted two further sets of exploratory analyses to compare our results with those by Boschloo et al. [96] and alterating computation of the SOrT metric to $\mathrm{SOrT}=\frac{\sum_i{m}_i{s}_i}{\sum_i{s}_i}$, so that there were no artifactual correlations with symptom severity (see Additional file 9 for details). Results suggested that symptom-based metrics from Boschloo et al. [96] and with altered SOrT score computation did not offer reliable advantages for allocation to psychotherapy versus ADM.

## Discussion

This registered report outlines a detailed investigation of the comparative effectiveness of psychotherapy and ADM for the treatment of individual depressive symptoms and whether symptom-specific effectiveness information can serve precision allocation. We did not find ADM or psychotherapy to be more effective than the respective other treatment at study endpoints in depressive symptom sum-scores on HAM-D and BDI scales. Similarly, there was no clear advantage of either treatment for individual depressive symptoms. Using individual symptom meta-analysis results, we evaluated the *Symptom-Oriented Therapy* (SOrT) metric, which combines meta-analytic effect size estimates with patients’ symptom profiles prior to treatment. Validation analyses in MARS and PReDICT studies did not indicate that the SOrT metric constituted a valid measure, nor that it should be used as a precision metric to indicate favourable allocation to psychotherapy versus ADM.

Our findings of comparable effectiveness of psychotherapy and ADM at the end of acute treatment closely align with older [40–42] and more recent [97] meta-analytic work. Cuijpers and colleagues only recently conducted an extensive network meta-analysis quantifying and ranking multiple different treatments for adult depression, including psychotherapy, ADM, and their combination [97, 98]: Across multiple sets of sensitivity analyses (e.g. high versus low risk of bias, optimised psychotherapy/ADM, excluding placebo-controlled studies) and looking at groups of studies with moderate, severe, and chronic depression, psychotherapy and ADM consistently showed comparable effectiveness. Yet, psychotherapy was more acceptable (as defined in terms of lower dropout rates) compared to ADM. It is reassuring that our findings, albeit with smaller number of included studies, fully replicate this report. Our exploratory meta-regression of the HAM-D further demonstrated that effectiveness estimates of specific studies were moderated by differential dropout in study arms. Accordingly, if only ADM *or* psychotherapy is available to patients (rather than the combination, which is most effective [97]), we agree with clinical recommendations by Cuijpers and colleagues in that psychotherapy would be preferable to ADM based on its greater acceptability. This indication may be particularly relevant for patients who are likely not to complete a full course of treatment and prone to dropout (e.g. younger patients [99]).

Symptom-specific meta-analyses did not show significant differential treatment effects of psychotherapy or ADM for specific depressive symptoms following multiple comparison corrections. Although we identified nominally significant treatment differences for some symptoms, these do not align with prior research findings [36, 38, 96]. Hence, it remains unclear whether true symptom-specific treatment differences exist or whether reports from our study and previous literature reflect false positive findings (see Additional file 10 for further discussion).

The absence of clear meta-analytic symptom differences may also explain why our proposed SOrT metric did not seem to offer any benefit for treatment allocation. Assuming meta-analytic effect sizes were representative of noise rather than signal, this would mean SOrT scores were merely superimposing (meta-analytic) noise onto patients’ baseline symptom profiles. Our failure to find predictive utility of the precision sum-score from Boschloo and colleagues [96] and of a SOrT metric based on their reported effect sizes adds to this discouraging conclusion that symptom scores may not be of value in predicting psychotherapy versus ADM response.

It is important to emphasise, however, that the SOrT metric and other symptom-based allocation metrics (cf. [96, 100]) are likely specific to the comparison being made, so are restricted to the comparison of psychotherapy versus ADM in this report. Khazanov and colleagues, for instance, recently showed that patients with greater distress and anhedonia prior to treatment responded more favourably to a combination of cognitive therapy and ADM rather than to ADM alone. This suggests individual symptom information may be valuable for indicating combination versus monotherapy in depression [100]. Similarly, there are reports that low-grade inflammation shows specificity for somatic symptoms of depression (e.g. sleep problems, low energy, or increased appetite) [101–104], so ongoing studies evaluating immunotherapy as a treatment for depression may benefit from symptom-based treatment allocation rules. We therefore encourage researchers to apply the SOrT metric to other treatment comparisons to evaluate its value as a precision medicine tool. It could be helpful, therefore, to adjust SOrT scores for symptom severity as we highlighted in our exploratory analyses (cf. Additional file 9). Moreover, future research could evaluate a combination of symptom-weighted approaches (such as the SOrT metric) with significance thresholds. Specifically, symptom-based metrics might benefit from only including meta-analytic weights (and corresponding symptoms) that pass a specific meta-analytic significance threshold for the comparison in question, thus optimising the signal-to-noise ratio. It is interesting to note that this approach would be similar to how polygenic risk scores are created (e.g. using PRSice software [105]), which weight effect alleles by effect sizes, if a specific significance threshold has been reached for this allele. In sum, we hope symptom-based precision medicine tools receive further attention in future research despite the limited value for decision-making on allocation to psychotherapy versus ADM in depression.

### Limitations

The present investigation has five major limitations. First, we were not able to include all studies identified from the literature in meta-analyses. This low coverage was due to varied reasons such as general data unavailability [59, 62, 63, 86, 89, 90], insufficient reporting of sample sizes and summary statistics for inclusion in sum-score meta-analysis [68, 75, 85, 94], and/or failure of authors to respond to inquiries (cf. Additional file 11: Table S19). Based on this, we encourage more thorough reporting of summary statistics in original trials (ideally including completer and intention-to-treat samples) and also advocate publication of summary statistics in meta-analyses rather than effect sizes alone; this would enhance reproducibility. Despite limited data availability, it is reassuring that conclusions from sum-score meta-analyses match the most recent meta-analysis with the largest power [97]. Sample sizes for individual symptom meta-analyses were also relatively large and exceeded those of similar work by Boschloo et al. [96], which can be attributed to our broader focus on RCTs comparing ADM with psychotherapy (rather than to CBT only).

Second, we cannot conclude anything regarding symptom-specific differences between intervention comparisons other than psychotherapy versus ADM (as discussed before), and it is unclear if conclusions outside this study’s inclusion criteria would be meaningful. Our inclusion criteria, for instance, focussed on acute treatment of adult depression, so it is possible that symptom-specific differences following treatment with ADM versus psychotherapy exist in children, adolescents, or older patients. Similarly, symptom-specific differences may arise at follow-up in the form of residual symptoms (also not addressed in this report), which was recently shown in a RCT re-analysis [38].

Third, our analyses focussed on study completer data, though an intention-to-treat approach would be clinically more interesting. The data format used for present analyses did not allow for the identification of individual participants, which facilitated data sharing from original RCTs. Due to non-identifiability of individuals, however, we were not able to use imputation techniques for intention-to-treat analyses on the symptom level. Consequently, we could not verify whether our findings would have been different if all randomised participants had been included, rendering this an open question for future research.

Fourth, inferences from symptom-specific analyses are limited by the low reliability of individual symptoms. Depression measures, such as the HAM-D and BDI used in the present report, are not designed for reliable symptom-specific assessments [32, 33]. Thus, our failure to find evidence for symptom-specific treatment effects and/or differences between present findings and Boschloo et al. [96] may be a consequence of low reliability of symptom assessments. Future research should ideally combine evidence from multiple indicators per assessed symptom to increase reliability.

Lastly, validation of the SOrT metric was only performed in data from the PReDICT study. Because the PReDICT study enrolled only treatment-naïve patients [44, 57], it may not have provided the optimal sample for validating the SOrT metric, which was derived from more mixed samples. Future research should thus evaluate the utility of symptom-based metrics in other samples, which would require individual patient data. Ideally, cross-validation procedures could be used, which would average over unique characteristics of multiple validation samples.

## Conclusion

In conclusion, we report the largest symptom-specific meta-analysis of direct comparisons of psychotherapy and ADM for depression. We did not find robust indications for symptom-specific effectiveness differences between treatments and this also extended to the sum-score level. We introduced the Symptom-Oriented Therapy (SOrT) metric as a precision treatment allocation tool, but failed to demonstrate its usefulness for improved allocation to psychotherapy or ADM. Though future symptom-specific work looking at other treatment comparisons could be valuable, our findings suggest that symptom information does not inform on whether any individual patient should receive psychotherapy or ADM.

## Supplementary information

**Additional file 1: Table S1.** Search Strategy for Identification of RCTs of Psychotherapy versus Pharmacotherapy for Depression.

**Additional file 2.** Standardised author contacting and quality evaluation procedure.

**Additional file 3 Tables S2-S4.** Content overlap assessment of Beck Depression Inventory (BDI) and Hamilton Rating Scale for Depression (HAM-D). Table S2- Content Overlap of HAM-D and BDI-II Items Sorted by Item Numbering. Table S3- Content Overlap of HAM-D and BDI-II Items Sorted by Equivalent Items. Table S4- Content Overlap of BDI-I and BDI-II Items Sorted by Item Numbering.

**Additional file 4: Table S5.** Development and considerations of a Symptom-Oriented Therapy (SOrT) metric. Table S5- Hypothetical SOrT Metric Computation for Three Patients.

**Additional file 5.** Timeline and timeline adherence of the registered report.

**Additional file 6: Table S6.** Study overview of RCTs included in qualitative synthesis.

**Additional file 7: Table S7, Figs. S1-S5.** Sum-score meta-analysis results. Table S7- Meta-regression of HAM-D and BDI sum-score meta-analyses on differential dropout. Fig. S1- Funnel plot of HAM-D sum-score meta-analysis. Fig. S2- Funnel plot of BDI sum-score meta-analysis. Fig. S3- Meta-Regression of HAM-D sum-score meta-analysis on differential dropout. Fig. S4- Meta-Regression of BDI sum-score meta-analysis on differential dropout. Fig. S5- Funnel plot of HAM-D sum-score meta-analysis. Fig. S6- Forest plot of dropout meta-analysis.

**Additional file 8: Tables S8-S10, Figs. S6-S10.** Individual symptom meta-analysis results and related sensitivity and exploratory analyses. Table S8- Individual symptom forest plots for the HAM-D. Table S9- Individual symptom forest plots for the BDI. Table S10- Correlations between meta-analytic effect size metrics. Fig. S6- Effect size comparison of HAM-D and BDI per symptom type. Fig. S7- Individual symptom effect sizes per effect size metric used in meta-analyses. Fig. S8- Associations between meta-analytic effect size metrics. Fig. S9- Effect size association to Boschloo et al. Fig. S10- Effect size association to Boschloo et al. for RCTs with CBT only.

**Additional file 9: Tables S11-S18, Fig. S11.** Validation analyses of SOrT metric in MARS and PReDICT samples and related exploratory analyses. Table S11- Effect sizes of individual symptom meta-analyses with versus without Dunlop et al. Table S12- Linear regression analysis of 12-week HAM-D sum-scores on SOrT-based BDI treatment allocation match following valence split. Table S13- Linear regression analysis of 12-week HAM-D sum-scores on SOrT-based treatment allocation match following median split. Table S14- Linear regression analysis of 12-week HAM-D sum-scores on SOrT-based treatment allocation match following 2/3 extreme group split. Table S15- Treatment allocation match prediction using Boschloo et al.-based SOrT and sum-scores. Table S16- Regression-based treatment allocation match prediction using Boschloo et al.-based SOrT and sum-scores. Table S17- Association of symptom severity with updated SOrT scores. Table S18- Treatment allocation match prediction using updated SOrT metric. Fig. S11- Association and distributions of HAM-D and BDI SOrT scores in PReDICT.

**Additional file 10.** Discussion on nominally significant treatment differences of psychotherapy and ADM for specific depressive symptoms.

**Additional file 11: Tables S19-S20.** Overview of available data and materials from original studies and presented work. Table S19 Availability of Original Study Data. Table S20. Online File Overview as Available on https://osf.io/qzjc9/.

## Data Availability

For maximum transparency and reproducibility, we attempt to provide as much data and materials as possible. In Additional file 11: Table S19, we outline to what capacity we can share data from original RCTs identified in the systematic review. In Additional file 11: Table S20, we provide a list of additional data and analysis scripts as available on the open science framework (OSF) platform under https://osf.io/qzjc9/. We are not able to publicly share original data from the MARS study. Original data from PReDICT, however, is available as required by NIH data sharing policy and can be requested by contacting original authors.

## Abbreviations

ADM: Antidepressant medication
BDI: Beck Depression Inventory
CBT: Cognitive behavioural therapy
CENTRAL: Cochrane Central Register of Controlled Trials
CI: Confidence interval
HAMA: Hamilton Anxiety Rating Scale
HAM-D: Hamilton Rating Scale for Depression
IPD: Individual patient data
MARS: Munich Antidepressant Response Signature
MDD: Major Depressive Disorder
PAI: Personalised Advantage Index
PReDICT: Emory Predictors of Remission in Depression to Individual and Combined Treatments
(p)OR: (Proportional) Odds ratio
RCT: Randomised controlled trial
SD: Standard deviation
SE: Standard error
SMD: Standardised mean difference
SOrT: Symptom-Oriented Therapy
