# Supplementary material for: Psychotherapy or medication for depression? Using individual symptom meta-analyses to derive a Symptom-Oriented Therapy (SOrT) metric for a personalised psychiatry
Source: BMC Med. 2020 Jun 5;18:170. doi: 10.1186/s12916-020-01623-9 (PMC7273646; doi:10.1186/s12916-020-01623-9)
Supplement: Supplementary file 1 — Additional file 1: Table S1. Search Strategy for Identification of RCTs of Psychotherapy versus Pharmacotherapy for Depression. [file 12916_2020_1623_MOESM1_ESM.docx]

**Additional file 1**

## Search Strategy and Study Selection

### Table S1: Search Strategy for Identification of RCTs of Psychotherapy versus Pharmacotherapy for Depression

| Database | Search |
| --- | --- |
| PubMed Medline | (depression[MeSH Terms] OR depressive disorder[MeSH Terms] OR mood disorder[MeSH Terms] OR affective* OR depress*) AND (psychotherapy [mh] OR (Cogniti* AND (technique* OR therap* OR restructur* OR challeng*)) AND (Antidepressive Agents [Pharmacological Action] OR agents, antidepressive[MeSH Terms] OR SSRI OR SNRI OR TCA OR "selective serotonin reuptake inhibitor" OR “selective norepinephrine inhibitor” OR “tricyclic antidepressant”) AND (randomized controlled trial[pt] OR controlled clinical trial[pt] OR randomized [tiab] OR randomly [tiab] OR trial [tiab] OR groups [tiab]) |
| CENTRAL | #1 MeSH descriptor: [Depression] explode all trees  #2 MeSH descriptor: [Mood Disorders] explode all trees  #3 MeSH descriptor: [Depressive Disorder] explode all trees  #4 affect* near disord*  #5 #1 OR #2 OR #3 OR #4  #6 MeSH descriptor [Psychotherapy] explode all trees  #7 Cogniti* near (technique* OR therap* OR restructur* OR challeng*)  #8 behavio* near therap*  #9 #6 OR #7 OR #8  #10 MeSH descriptor [Antidepressive Agents] explode all trees  #11 SSRI OR SNRI OR TCA OR "selective serotonin reuptake inhibitor" OR “selective norepinephrine inhibitor” OR “trycyclic antidepressant”  #12 #10 OR #11  #13 #5 AND #9 AND #12 |
| PsychINFO | #1 exp MAJOR DEPRESSION/ or exp ZUNGS SELF RATING DEPRESSION SCALE/ or exp "DEPRESSION (EMOTION)"/ or exp POSTPARTUM DEPRESSION/ or exp SPREADING DEPRESSION/ or exp BECK DEPRESSION INVENTORY/ or exp RECURRENT DEPRESSION/ or exp ATYPICAL DEPRESSION/ or exp LATE LIFE DEPRESSION/ or exp ANACLITIC DEPRESSION/ or exp TREATMENT RESISTANT DEPRESSION/ or exp "LONG-TERM DEPRESSION (NEURONAL)"/  #2 exp PSYCHOTHERAPY/  #3 exp Antidepressant Drugs/  #4 1 and 2 and 3  #5 Treatment Effectiveness Evaluation/  #6 exp PLACEBO/  #7 exp Treatment Outcomes/  #8 exp Followup Studies/  #9 placebo*.mp. [mp=title, abstract, heading word, table of contents, key concepts, original title, tests & measures]  #10 random*.mp. [mp=title, abstract, heading word, table of contents, key concepts, original title, tests & measures]  #11 comparative stud*.mp. [mp=title, abstract, heading word, table of contents, key concepts, original title, tests & measures]  #12 "clinical NEAR/3 trial*".mp. [mp=title, abstract, heading word, table of contents, key concepts, original title, tests & measures]  #13 "NEAR/3 design".mp. [mp=title, abstract, heading word, table of contents, key concepts, original title, tests & measures]  #14 "evaluat* NEAR/3 stud*".mp. [mp=title, abstract, heading word, table of contents, key concepts, original title, tests & measures]  #15 "prospectiv* NEAR/3 stud*".mp. [mp=title, abstract, heading word, table of contents, key concepts, original title, tests & measures]  #16 "(singl* OR doubl* OR trebl* ORtripl*) NEAR/3 (blind* OR mask*)".mp. [mp=title, abstract, heading word, table of contents, key concepts, original title, tests & measures]  #17 5 or 6 or 7 or 8 or 9 or 10 or 11 or 12 or 13 or 14 or 15 or 16  #18 4 and 17 |
