# Supplementary material for: Psychotherapy or medication for depression? Using individual symptom meta-analyses to derive a Symptom-Oriented Therapy (SOrT) metric for a personalised psychiatry
Source: BMC Med. 2020 Jun 5;18:170. doi: 10.1186/s12916-020-01623-9 (PMC7273646; doi:10.1186/s12916-020-01623-9)
Supplement: Supplementary file 2 — Additional file 2. Standardised author contacting and quality evaluation procedure. [file 12916_2020_1623_MOESM2_ESM.docx]

**Additional file 2**

**Data acquisition and extraction: Standardised author contacting and quality evaluation procedure**

First, we emailed corresponding authors with a request to provide data. If we did not get a response within one month or if we could not obtain contact information for the corresponding author, we tried to contact other study co-authors and sent a reminder to the corresponding author. This procedure was followed until a maximum of three attempts of contacting each author were reached. Data were classified as unavailable when authors did not respond to multiple contact attempts or if contacted authors stated that they do not have access to the data anymore. After receiving the data, we performed an initial check for the concordance between the received data and the data reported in the published papers before including them in the meta-analyses. The data were examined regarding sample sizes and means and standard deviations of outcome variables. When discrepancies arose, original authors were approached for clarification. If clarification was not possible, the impact of the differences were evaluated by NK and JKB as to whether any biases would result for the overall results from the present investigation.
