# Supplementary material for: Psychotherapy or medication for depression? Using individual symptom meta-analyses to derive a Symptom-Oriented Therapy (SOrT) metric for a personalised psychiatry
Source: BMC Med. 2020 Jun 5;18:170. doi: 10.1186/s12916-020-01623-9 (PMC7273646; doi:10.1186/s12916-020-01623-9)
Supplement: Supplementary file 3 — Additional file 3 Tables S2-S4. Content overlap assessment of Beck Depression Inventory (BDI) and Hamilton Rating Scale for Depression (HAM-D). Table S2- Content Overlap of HAM-D and BDI-II Items Sorted by Item Numbering. Table S3- Content Overlap of HAM-D and BDI-II Items Sorted by Equivalent Items. Table S4- Content Overlap of BDI-I and BDI-II Items Sorted by Item Numbering. [file 12916_2020_1623_MOESM3_ESM.docx]

**Additional file 3**

## Statistical Analysis

### Table S2: Content Overlap of HAM-D and BDI-II Items Sorted by Item Numbering

| BDI |  |  |  | HAM-D |
| --- | --- | --- | --- | --- |
| Item number and title | Item content |  | Item content | Item number and title |
| 1. Sadness | - **Sadness** - **Unhappiness** |  | - **Gloomy** - **Pessimism** - **Weeping** - **Sadness** | 1. Depressed mood |
| 1. Pessimism | - **Pessimism** - **Hopelessness** |  | - **Guilt** - **Punishment** | 1. Feelings of guilt |
| 1. Past Failure | - **4 stages of personal failure** |  | - **4 different stages** | 1. Suicide |
| 1. Loss of pleasure | - **4 stages of loss of pleasure** |  | - **2 different stages** | 1. Insomnia (initial) |
| 1. Guilty feelings | - **4 stages of feelings of guilty** |  | - **2 different stages** | 1. Insomnia (middle) |
| 1. Punishment feelings | - **4 stages of feelings of punishment** |  | - **2 different stages** | 1. Insomnia (delayed) |
| 1. Self-Dislike | - **Lost confidence** - **Disappointment** - **Dislike** |  | - **Incapacity** - **Indecision** - **Listlessness** - **Loss of interest in hobbies and social activities** - **Decreased productivity** - **Unable to work** | 1. Work and interest |
| 1. Self-Criticism | - **4 stages of self-criticism** |  | - **Retardation** - **Stupor** | 1. Retardation |
| 1. Suicidal thoughts and whishes | - **4 stages of willingness to kill oneself** |  | - **Agitation** | 1. Agitation |
| 1. Crying | - **4 stages of crying** |  | - **Tension** - **Irritability** - **Worrying** - **Fears** | 1. Anxiety (psychic) |
| 1. Agitation | - **Restlessness** - **Agitation** |  | - **4 different stages** | 1. Anxiety (somatic) |
| 1. Loss of interest | - **Loss of interest in social environment** - **Loss of general interest** |  | - **Loss of appetite** - **Feeling in abdomen** - **Constipation** | 1. Somatic symptoms (gastrointestinal) |
| 1. Indecisiveness | - **4 stages of Indecisiveness** |  | - **Heaviness in limbs, head, or back** - **Loss energy** - **Fatigability** - **2 stages** | 1. Somatic symptoms (general) |
| 1. Worthlessness | - **4 stages of feeling worthless** |  | - **Loss of libido** - **Menstrual disturbances** - **2 stages** | 1. Genital symptoms |
| 1. Loss of energy | - **4 stages of loss of energy** |  | - **4 stages from**   **Self-absorption to delusions** | 1. Hypochondriasis |
| 1. Change in sleeping pattern | - **Differentiation between more or less sleep** - **3 different stages** |  | - **3 stages of loss of insight** | 1. Insight |
| 1. Irritability | - **4 stages of irritability** |  | - **2 stages of weight loss** | 1. Weight loss |
| 1. Changes in appetite | - **Differentiation between more or less appetite** - **3 different stages** |  |  |  |
| 1. Concentration difficulty | - **4 stages of concentration difficulties** |  |  |  |
| 1. Tiredness or fatigue | - **4 stages of fatigue** |  |  |  |
| 1. Loss of interest in sex | - **4 stages of loss of libido** |  |  |  |

### Table S3: Content Overlap of HAM-D and BDI-II Items Sorted by Equivalent Items

| BDI |  |  |  | Hamilton |
| --- | --- | --- | --- | --- |
| Item number and title | Item content |  | Item content | Item number and title |
| **BDI-items with an HAM-D equivalent** | | | | |
| Sadness (1) | - **Sadness** - **Unhappiness** |  | - **Sadness** - **Gloomy** - **Pessimism** - **Weeping** | Depressed mood (1) |
| Pessimism (2) | - **Pessimism** - **Hopelessness** |  |  |  |
| Crying (10) | - **4 stages of crying** |  |  |  |
| Guilty feelings (5) | - **4 stages of feelings of guilty** |  | - **Guilt** - **Punishment** | Feelings of guilt (2) |
| Punishment feelings (6) | - **4 stages of feelings of punishment** |  |  |  |
| Suicidal thoughts and whishes (9) | - **4 stages of willingness to kill oneself** |  | **4 different stages** | Suicide (3) |
| Agitation (11) | - **Restlessness** - **Agitation** |  | - **Agitation** | Agitation (9) |
| Indecisiveness (13) | - **4 stages of Indecisiveness** |  | - **Incapacity** - **Indecision** - **Listlessness** - **Loss of interest in hobbies and social activities** - **Decreased productivity** - **Unable to work** | Work and interest (7) |
| Loss of interest (12) | - **Loss of interest in social environment** - **Loss of general interest** |  |  |  |
| Loss of energy (15) | - **4 stages of loss of energy** |  | - **Heaviness in limbs, head, or back** - **Loss energy** - **Fatigability** - **2 stages** | Somatic symptoms (general) (13) |
| Tiredness or fatigue (20) | - **4 stages of fatigue** |  |  |  |
| Loss of interest in sex (21) | - **4 stages of loss of libido** |  | - **Loss of libido** - **Menstrual disturbances**   **2 stages** | Genital symptoms (14) |
| **BDI-items with an HAM-D semi equivalent** | | | | |
| Irritability (17) | - **4 stages of irritability** |  | - **Tension** - **Irritability** - **Worrying** - **Fears** | Anxiety (psychic) (10) |
| Change in sleeping pattern (16) | - **Differentiation between more or less sleep** - **3 different stages** |  | - **2 different stages** | Insomnia (initial) (4) |
|  |  |  | - **2 different stages** | Insomnia (middle) (5) |
|  |  |  | - **2 different stages** | Insomnia (delayed) (6) |
| Changes in appetite (18) | - **Differentiation between more or less appetite** - **3 different stages** |  | - **Loss of appetite** - **Feeling in abdomen** - **Constipation** | Somatic symptoms (gastrointestinal) (12) |

| **BDI-items with no HAM-D equivalent** | | | | |
| --- | --- | --- | --- | --- |
| Past Failure (3) | - **4 stages of personal failure** |  |  |  |
| Loss of pleasure (4) | - **4 stages of loss of pleasure** |  |  |  |
| Self-Dislike (7) | - **Lost confidence** - **Disappointment** - **Dislike** |  |  |  |
| Self-Criticism (8) | - **4 stages of self-criticism** |  |  |  |
| Worthlessness (14) | - **4 stages of feeling worthless** |  |  |  |
| Concentration difficulty (19) | - **4 stages of concentration difficulties** |  |  |  |
| **HAMD-items with no BDI equivalent** | | | | |
|  |  |  | - **Retardation** - **Stupor** | Retardation (8) |
|  |  |  | **4 different stages** | Anxiety (somatic) (11) |
|  |  |  | - **4 stages from**   **Self-absorption to delusions** | Hypochondriasis (15) |
|  |  |  | **3 stages of loss of insight** | Insight (16) |
|  |  |  | **2 stages of weight loss** | Weight loss (17) |

### Table S4: Content Overlap of BDI-I and BDI-II Items Sorted by Item Numbering

| Item number | BDI-I | Item number | BDI-II | Equivalency |
| --- | --- | --- | --- | --- |
| A | mood | 1 | sadness | similar enough for aggregation |
| B | pessimism | 2 | pessimism | similar enough for aggregation |
| C | sense of failure | 3 | past failure | similar enough for aggregation |
| D | lack of satisfaction | 4 | loss of pleasure | similar enough for aggregation |
| E | guilty feeling | 5 | guilty feelings | similar enough for aggregation |
| F | sense of punishment | 6 | punishment feelings | similar enough for aggregation |
| G | self-hate | 7 | self-dislike | similar enough for aggregation |
| H | self-accusation | 8 | self-criticalness | similar enough for aggregation |
| I | self-punitive wishes | 9 | suicidal thoughts or wishes | similar enough for aggregation |
| J | crying spells | 10 | crying | similar enough for aggregation |
| K | irritability | 11 | agitation | BD-I: K similar enough for aggregation to BDI-II: 17 |
| L | social withdrawal | 12 | loss of interest | similar enough for aggregation |
| M | indecisiveness | 13 | indecisiveness | similar enough for aggregation |
| N | body image | 14 | worthlessness | different |
| O | work inhibition | 15 | loss of energy | different |
| P | sleep disturbance | 16 | changes in sleeping pattern | similar enough for aggregation |
| Q | fatigability | 17 | irritability | BDI-I: Q similar enough for aggregation to BDI-II: 20 |
| R | loss of appetite | 18 | changes in appetite | similar enough for aggregation |
| S | weight loss | 19 | concentration difficulty | different |
| T | somatic preoccupation | 20 | tiredness or fatigue | BDI-II: 20 similar enough for aggregation to BDI-I: Q |
| U | loss of libido | 21 | loss of interest in sex | equivalent |
