# Supplementary material for: Psychotherapy or medication for depression? Using individual symptom meta-analyses to derive a Symptom-Oriented Therapy (SOrT) metric for a personalised psychiatry
Source: BMC Med. 2020 Jun 5;18:170. doi: 10.1186/s12916-020-01623-9 (PMC7273646; doi:10.1186/s12916-020-01623-9)
Supplement: Supplementary file 4 — Additional file 4: Table S5. Development and considerations of a Symptom-Oriented Therapy (SOrT) metric. Table S5- Hypothetical SOrT Metric Computation for Three Patients. [file 12916_2020_1623_MOESM4_ESM.docx]

# Additional file 4

## Development of a Symptom-Oriented Therapy (SOrT) Metric

The SOrT metric aims to provide a quantitative suggestion on whether patients will profit more from ADM or psychotherapy (or not) based on their individual symptom profile. This is why patients’ depressive symptoms (*s* parameter in formula) will weigh meta-analytic effect sizes (*m* parameter in formula) on an individual symptom basis (*i* parameter in formula) using the formula $SOrT=\sum_{i} {m_{i}s}_{i}$. Since *m* is a meta-analysis based effect size as a standardised mean difference (SMD), it is centred at 0 and favours ADM or psychotherapy with increasing negative or positive values, respectively (depending on coding used in meta-analysis). This means individual symptom scores can be considered as weights that “push” the treatment suggestion (as offered by meta-analysis) in the direction of either treatment. For example, a patient who only expresses symptoms better treated by ADM (as suggested by our meta-analysis)- but not symptoms better treated by psychotherapy- would receive a SOrT score favouring ADM. Online Supplementary Table 5 shows this for hypothetical patient scenarios with three symptoms. Here, SOrT scores would indicate preference of psychotherapy for patient 1, preference of ADM for patient 2, and no preference for either treatment for patient 3.

The SOrT metric is a parsimonious means of quantifying treatment preference based on our results and the patient symptom profile. While its simplicity makes the SOrT metric easy to use, this might leave out other important clinical information. For instance, patients’ (or clinicians’) opinion on the relevance of individual symptoms could be a very important additional parameter for weighing of the SOrT score (e.g., using coding: 0=not relevant; 0.5=relevant; 1=highly relevant). An alternative idea could be to include an evidence-based parameter for symptom relevance, for instance, with regards to impairment in psychosocial functioning. Regarding impairment of psychosocial functioning, Fried and Nesse have recently estimated symptom relevance for symptoms of the Quick Inventory of Depressive Symptoms (QIDS) in patients participating in the STAR*D study [39]. Either of these ideas could be implemented as another parameter (e.g., *r* for relevance) in the formula to weigh the score by the symptom relevance: $SOrT=\sum_{i} {m_{i}s}_{i}r_{i}$. Future research could apply such ratings and see whether the addition of, for example, a relevance parameter *r* has implications for treatment allocation, success, or with regards to other clinical correlates.

Lastly, we want to note that the SOrT metric would offer a starting point for treatment differentiation (and potentially treatment allocation) but still neglects other dimensions that need to be addressed in the future. For instance, an individual patient’s SOrT score would give a *suggestion* on whether his/ her symptoms are better treated by psychotherapy, ADM, or neither. It does not, however, offer guidance on whether any of these treatments should be used or a combination of treatments (i.e., ADM *and* psychotherapy), which is common in clinical practice. We chose to compare ADM with psychotherapy to maximise the qualitative treatment differences and to “prove” (or disprove) the concept of the SOrT metric. If our results showed support for utility of the SOrT metric, a next step could be the development of a distinct (or supplementary) metric to differentiate use of single or combination of treatments, for instance.

### Table S5: Hypothetical SOrT Metric Computation for Three Patients

|  | m^a^ | Patient 1 | Patient 2 | Patient3 |
| --- | --- | --- | --- | --- |
| s_1_ | 0.2 | 3 | 1 | 2 |
| s_2_ | 0.1 | 0 | 2 | 2 |
| s_3_ | -0.2 | 2 | 3 | 3 |
| SOrT Score |  | 0.2 | -0.2 | 0 |

^a^ *m* is the meta-analysis weighted effect size with positive values favouring psychotherapy and negative values favouring ADM.
