# Supplementary material for: Psychotherapy or medication for depression? Using individual symptom meta-analyses to derive a Symptom-Oriented Therapy (SOrT) metric for a personalised psychiatry
Source: BMC Med. 2020 Jun 5;18:170. doi: 10.1186/s12916-020-01623-9 (PMC7273646; doi:10.1186/s12916-020-01623-9)
Supplement: Supplementary file 5 — Additional file 5. Timeline and timeline adherence of the registered report. [file 12916_2020_1623_MOESM5_ESM.docx]

**Additional file 5**

## Timeline

After pre-acceptance of this registered report, we immediately started with the systematic review following specified search strategy. We estimated two months from screening articles in databases to the final decision of exclusion or inclusion. Next, we had two rounds of attempts (one month each) to contact authors of included studies with requests to share individual symptom data. At the same time as contacting authors, we also already started to read and evaluate included studies and extract specified data, which we estimated would take around four months (two of which overlap with the time required to contact authors). After we had allowed sufficient time to send us the data, we processed and analysed data on the individual symptom level and computed and evaluated the SOrT metric using MARS and PReDICT data, which we thought would require two months. After completion of meta-analyses and study evaluation, we required another two to four months to aggregate results and finalise the manuscript. In sum, we thus thought we would require around 10-12 months to move from pre-acceptance of the registered report to submission of a final manuscript.

## Timeline Adherence

We deviated with three months from the initial timeline of one year from pre-acceptance to stage 2 submission of the registered report. This deviation and non-adherence was caused by unforeseen changes outside of the project that resulted in postponement of decisions on study inclusions, contacting of original authors, and corollary tasks.
