# Supplementary material for: Psychotherapy or medication for depression? Using individual symptom meta-analyses to derive a Symptom-Oriented Therapy (SOrT) metric for a personalised psychiatry
Source: BMC Med. 2020 Jun 5;18:170. doi: 10.1186/s12916-020-01623-9 (PMC7273646; doi:10.1186/s12916-020-01623-9)
Supplement: Supplementary file 6 — Additional file 6: Table S6. Study overview of RCTs included in qualitative synthesis. [file 12916_2020_1623_MOESM6_ESM.docx]

**Additional file 6**

## Step 1: Systematic Review and Meta-analysis of RCTs of ADM versus Psychotherapy

## Literature Search

### Table S6: Study overview of RCTs included in qualitative synthesis

|  |  | Psychotherapy |  | ADM |  |  |  | SMD^†^ |  |
| --- | --- | --- | --- | --- | --- | --- | --- | --- | --- |
| Study | Country | Type | Sample size | Type | Sample size | Study Duration | Question-naires | HAM-D | BDI |
| Barber *et al.* (2012) | US | Supportive-expressive therapy | 39 | Protocol with sertraline switched to venlafaxine | 33 | 16 weeks | HAM-D | 0.03 | - |
| Bastos *et al.* (2013, 2015) | Brazil | LTPP | 73 | fluoxetine | 67 | 104 weeks | BDI-II | - | -0.87 |
| Bedi *et al.* (2000) | UK | Non-standardised counselling | 39 | ADM of choice | 44 | 8 weeks | BDI-I | - | 0.03 |
| Blackburn *et al.* (1981) | UK | CT | 22 | ADM of choice (usually amitriptyline or clomipramine) | 20 | Not fixed; Mean of 12.5 weeks | BDI-I & HAM-D | NA | NA |
| Blackburn *et al.* (1997) | UK | CT | 24 | ADM of choice | 43 | 16 weeks | BDI-I & HAM-D | -0.14 | -0.13 |
| Blom *et al.* (2007) | Netherlands | IPT | 34 | nefazodone | 30 | 12-16 weeks | HAM-D | -0.03 | - |
| David *et al.* (2008) | Romania | - CT - REBT | - 50 - 52 | fluoxetine | 49 | 14 weeks | BDI-I & HAM-D | -0.01 | -0.06 |
| Dekker *et al.* (2008) | Netherlands | STPP | 38 | venlafaxine | 34 | 8 weeks | HAM-D | 0.22 | - |
| DeRubeis *et al.* (2005) | US | CT | 51 | Protocol of paroxetine augmented with lithium carbonate or desipramine hydrochloride | 101 | 16 weeks | HAM-D | NA | - |
| DiMascio *et al.* (1979) | US | Short-term IPT | 12 | amitriptyline | 8 | 16 weeks | HAM-D | NA | - |
| Dimidjian *et al.* (2006) | US | - BA - CT | - 36 - 39 | paroxetine | 56 | 16 weeks | BDI-II & HAM-D | -0.04 | 0.40 |
| Dunlop *et al.* (2017) | US | CBT | 69 | - Escitalopram - duloxetine | - 86 - 79 | 12 weeks | BDI-II & HAM-D | 0.18 | 0.22 |
| Elkin *et al.* (1989) | US | - CBT - IPT | - 37 - 47 | Imipramine hydrochloride | 37 | 16 weeks | BDI-I & HAM-D | 0.03 | 0.28 |
| Frank *et al.* (2011) | US & Italy | IPT | 130 | escitalopram | 122 | 6 weeks | HAM-D | - | - |
| Harkness *et al.* (2012) | Canada | - CBT - IPT | - 47 - 50 | protocol | 43 | 16 weeks | HAM-D | 0.36 | - |
| Hollon *et al.* (1992) | US | CT | 16 | Imipramine hydrochloride | 32 | 12 weeks | BDI-I & HAM-D | 0.05 | -0.25 |
| Husain *et al.* (2014) | Pakistan | Group psychosocial treatment | 32 | fluoxetine | 32 | 3 months | HAM-D | 0.00 | - |
| Jarrett *et al.* (1999) | US | CT | 36 | phenelzine | 36 | 10 weeks | BDI-I & HAM-D | 0.19 | 0.21 |
| Keller *et al.* (2000) | US | CBASP | 156 | nefazodone | 153 | 12 weeks | HAM-D | 0.03 | - |
| Kennedy *et al.* (2007) | Canada | CBT | 12 | venlafaxine | 12 | 16 weeks | HAM-D | 0.31 | - |
| Lopez Rodriguez *et al.* (2004) | Mexico | Bellak psychotherapy | NA | fluoxetine | NA | 6 months | HAM-D | - | - |
| Martin *et al.* (2001) | UK | IPT | 13 | venlafaxine | 15 | 6 weeks | BDI-I & HAM-D | 0.55 | 0.51 |
| McGrath *et al.* (2013) | US | CBT | 34 | escitalopram | 33 | 12 weeks | BDI-II & HAM-D | 0.08 | 0.19 |
| McKnight *et al.* (1992) | US | CT | 22 | TCA | 21 | 8 weeks | BDI-I | - | 0.46 |
| McLean *et al.* (1979) | Canada | - STPP - Behaviour therapy | - 37 - 40 | amitriptyline | 39 | 10 weeks | BDI-I | - | NA |
| Menchetti *et al.* (2014) | Italy | Interpersonal counselling | 130 | SSRI (sertraline or citalopram) | 126 | 2 months | HAM-D | -0.11 | - |
| Miranda *et al.* (2003) | US | CBT | NA | Protocol with paroxetine potentially switched to buproprion | NA | No precise duration reported | BDI & HAM-D | NA | NA |
| Moradveisi *et al.* (2013) | Iran | BA | 45 | sertraline | 35 | 12 weeks | BDI-II & HAM-D | -0.63 | -0.70 |
| Murphy *et al.* (1995) | US | CBT | 11 | Desipramine hydrochloride | 7 | 16 weeks | BDI-I & HAM-D | -1.14 | -0.84 |
| Mynors-Wallis *et al.* (1995) | UK | Problem-solving therapy | 29 | amitriptyline | 27 | 12 weeks | BDI-I & HAM-D | -0.13 | -0.26 |
| Mynors-Wallis *et al.* (2000) | UK | Problem-solving therapy | 57 | SSRI (paroxetine or fluvoxamine) | 30 | 12 weeks | BDI-I & HAM-D | 0.35 | 0.08 |
| Parker *et al.* (2013) | Australia | CBT | 11 | ADM protocol | 18 | 12 weeks | HAM-D | 0.58 | - |
| Rush *et al.* (1977) | US | CT | 18 | Imipramine | 14 | 12 weeks | BDI-I & HAM-D | -0.51 | -0.73 |
| Salminen *et al.* (2008) | Finland | STPP | 21 | Fluoxetine | 19 | 16 weeks | BDI-II & HAM-D | 0.00 | -0.055 |
| Scott & Freeman (1992) | UK | - CBT - Social work counselling | - 29 - 29 | Amitriptyline | 26 | 16 weeks | HAM-D | -0.33 | - |
| Shamsaei *et al.* (2009) | Iran | CT | NA | citalopram | NA | 8 weeks | BDI-II | - | NA |
| Thompson *et al.* (2001) | US | CBT | 31 | Desipramine | 33 | 16-20 weeks | BDI & HAM-D | -0.21 | -0.15 |
| Zu *et al.* (2014) | China | CBT | 12 | ADM of choice | 25 | 24 weeks | HAM-D | -0.66 | - |

*Note:* Sample sizes refer to end-of-treatment completers. ^†^SMD: Positive values favour ADM while negative values favour PT. Abbreviations: BA=behavioural activation; BDI= Beck Depression Inventory; CBASP=Cognitive Behavioural Analysis System of Psychotherapy; CBT=Cognitive Behavioural Psychotherapy; CT=Cognitive Therapy; HAM-D=Hamilton Depression Rating Scale; IPT=interpersonal psychotherapy; LTPP=Long-term psychodynamic psychotherapy; NA=not available; REBT=rational emotive behaviour therapy; STPP=Short-term psychodynamic psychotherapy; TCA=tricyclic antidepressant medication
