# Supplementary material for: Psychotherapy or medication for depression? Using individual symptom meta-analyses to derive a Symptom-Oriented Therapy (SOrT) metric for a personalised psychiatry
Source: BMC Med. 2020 Jun 5;18:170. doi: 10.1186/s12916-020-01623-9 (PMC7273646; doi:10.1186/s12916-020-01623-9)

**Additional file 7**

## Sum-Score Meta-analyses

### Figure S1: Funnel plot of HAM-D sum-score meta-analysis


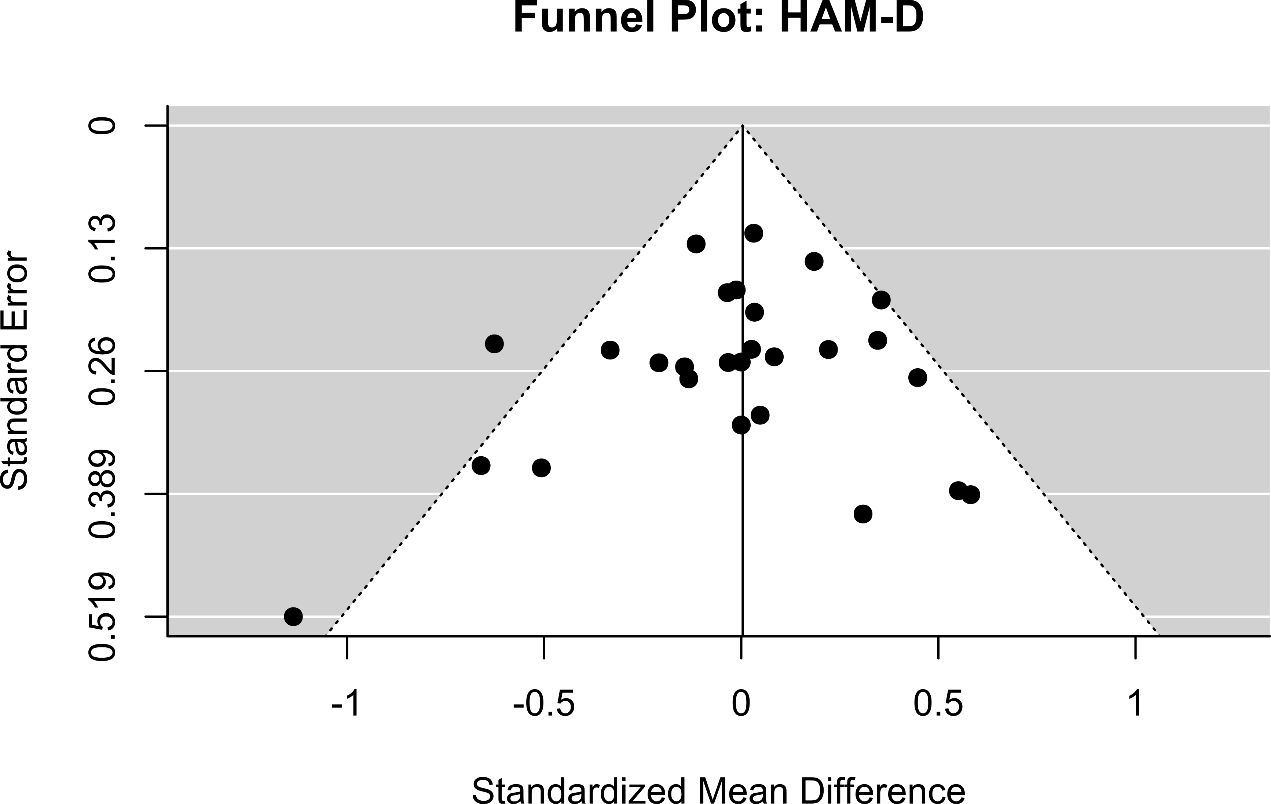


### Figure S2: Funnel plot of BDI sum-score meta-analysis


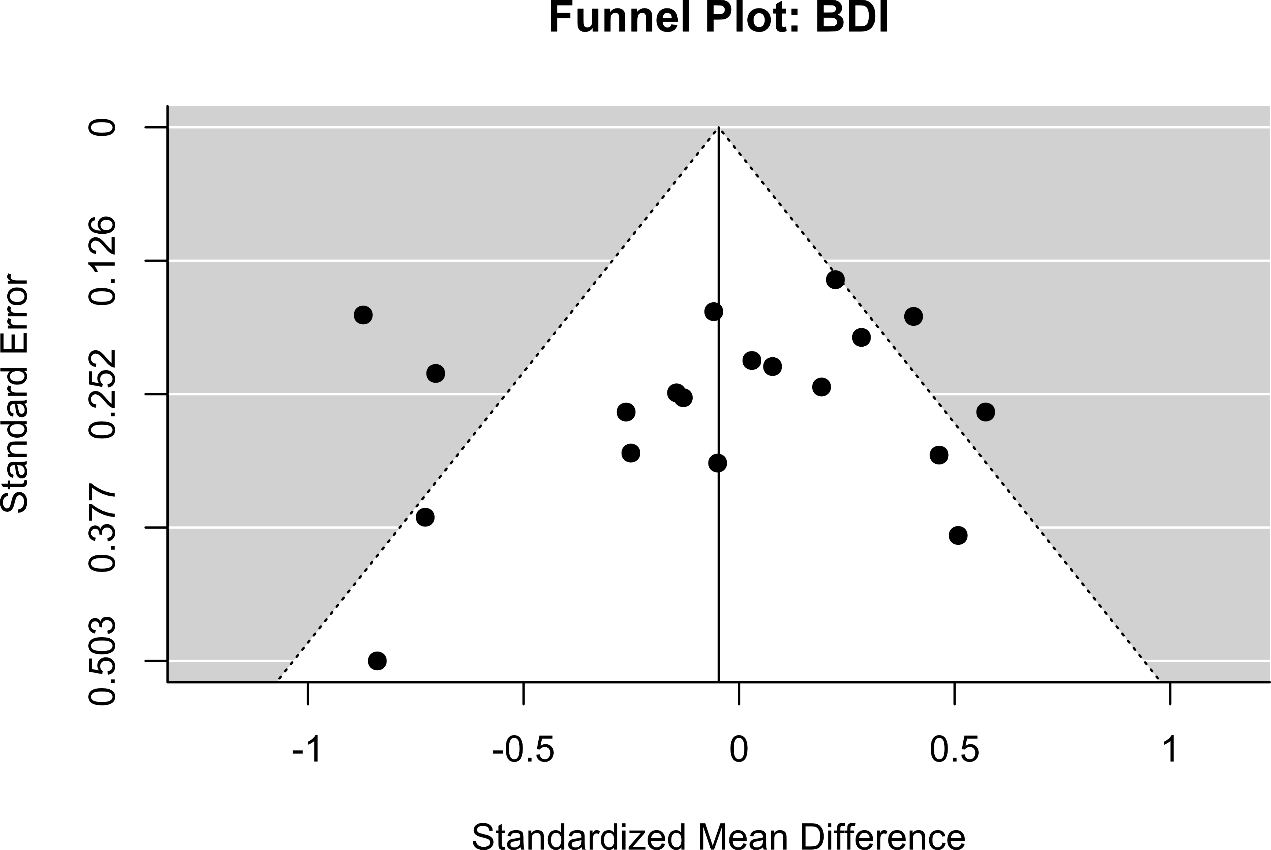


### Table S7: Meta-regression of HAM-D and BDI sum-score meta-analyses on differential dropout

|  | HAM-D | | BDI | |
| --- | --- | --- | --- | --- |
|  | Beta (SE) | P | Beta (SE) | P |
| Intercept | 0.060 (0.052) | 0.254 | 0.030 (0.107) | 0.782 |
| Moderator† | -0.011 (0.004) | 0.018 | -0.012 (0.007) | 0.124 |

*Note*: †Moderator is differential dropout as percentage of greater dropout in ADM arm(s) versus psychotherapy arm(s).

### Figure S3: Meta-Regression of HAM-D sum-score meta-analysis on differential dropout


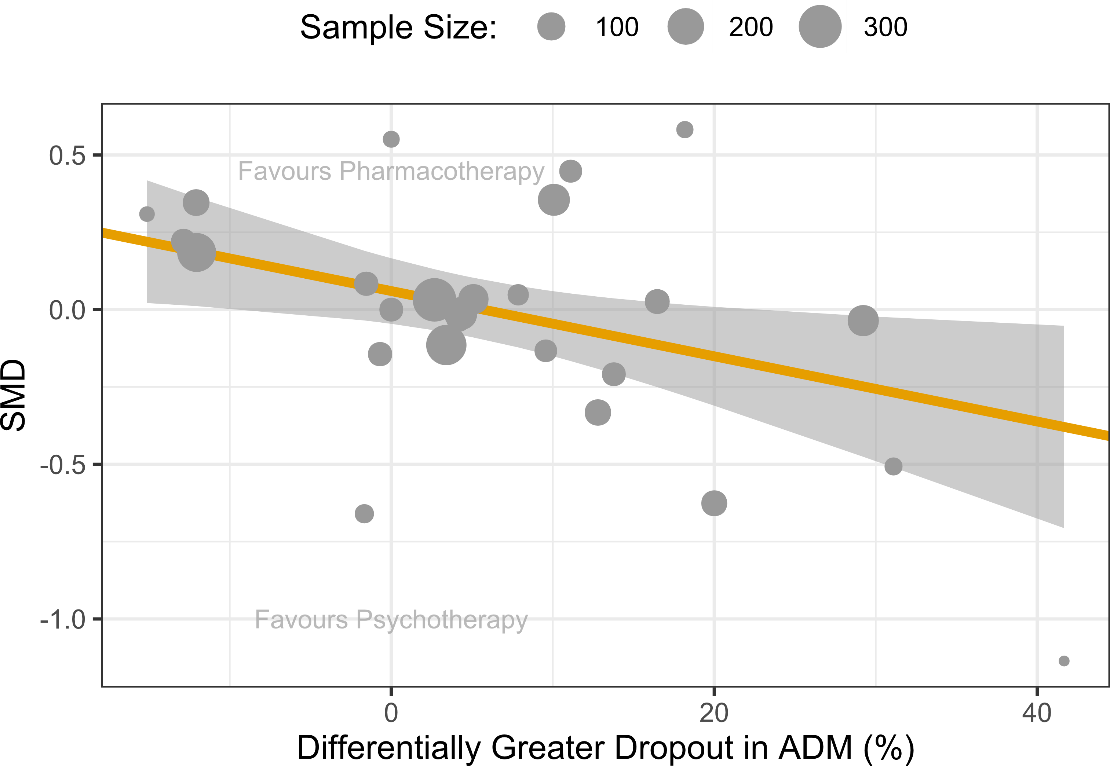


*Note*: Shaded area highlights 95% CI as predicted from meta-regression model.

### Figure S4: Meta-Regression of BDI sum-score meta-analysis on differential dropout


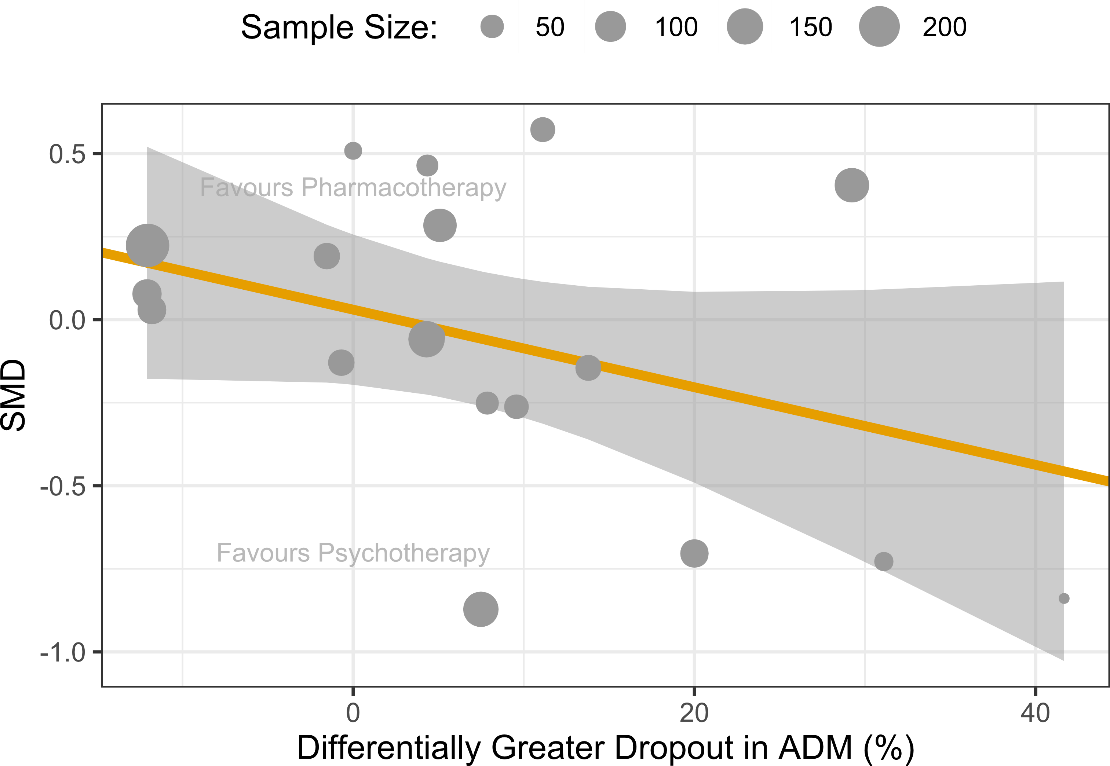


*Note*: Shaded area highlights 95% CI as predicted from meta-regression model.

### Figure S5: Forest plot of dropout meta-analysis


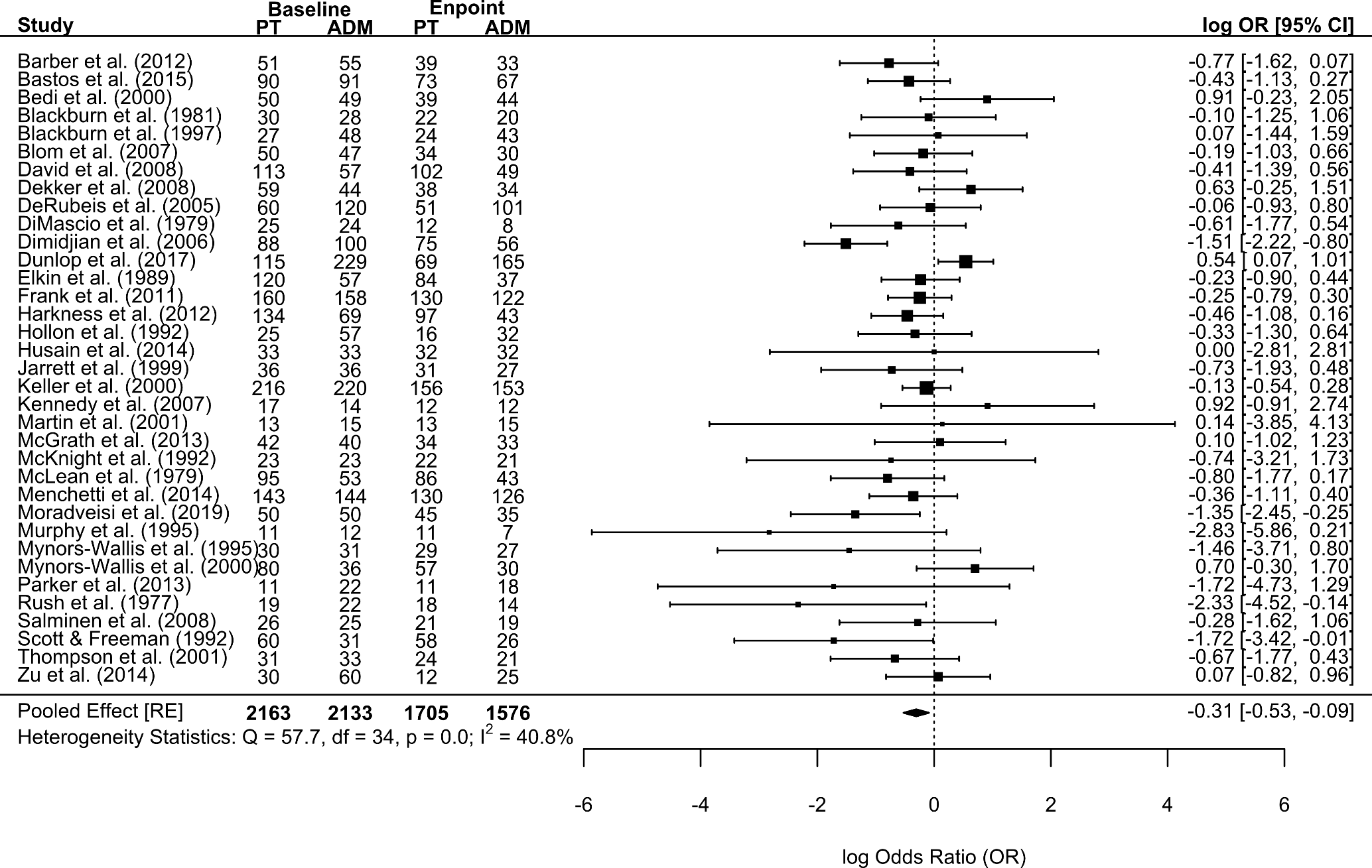

Supplement: Supplementary file 7 — Additional file 7: Table S7, Figs. S1-S5. Sum-score meta-analysis results. Table S7- Meta-regression of HAM-D and BDI sum-score meta-analyses on differential dropout. Fig. S1- Funnel plot of HAM-D sum-score meta-analysis. Fig. S2- Funnel plot of BDI sum-score meta-analysis. Fig. S3- Meta-Regression of HAM-D sum-score meta-analysis on differential dropout. Fig. S4- Meta-Regression of BDI sum-score meta-analysis on differential dropout. Fig. S5- Funnel plot of HAM-D sum-score meta-analysis. Fig. S6- Forest plot of dropout meta-analysis. [file 12916_2020_1623_MOESM7_ESM.docx]
