# Supplementary material for: Psychotherapy or medication for depression? Using individual symptom meta-analyses to derive a Symptom-Oriented Therapy (SOrT) metric for a personalised psychiatry
Source: BMC Med. 2020 Jun 5;18:170. doi: 10.1186/s12916-020-01623-9 (PMC7273646; doi:10.1186/s12916-020-01623-9)

**Additional file 8**

## Individual Symptom Meta-analyses

### Table S8: Individual symptom forest plots for the HAM-D

| HAM-D symptom | Forest Plot |
| --- | --- |
| 1 | 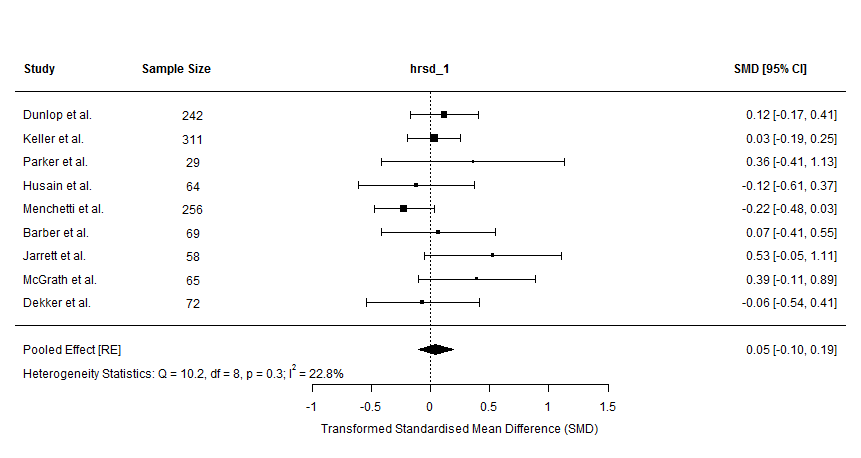 |
| 2 | 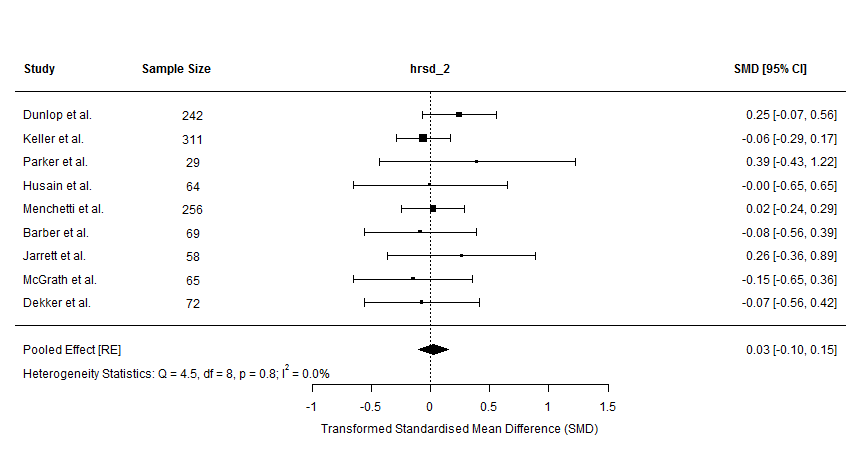 |
| 3 | 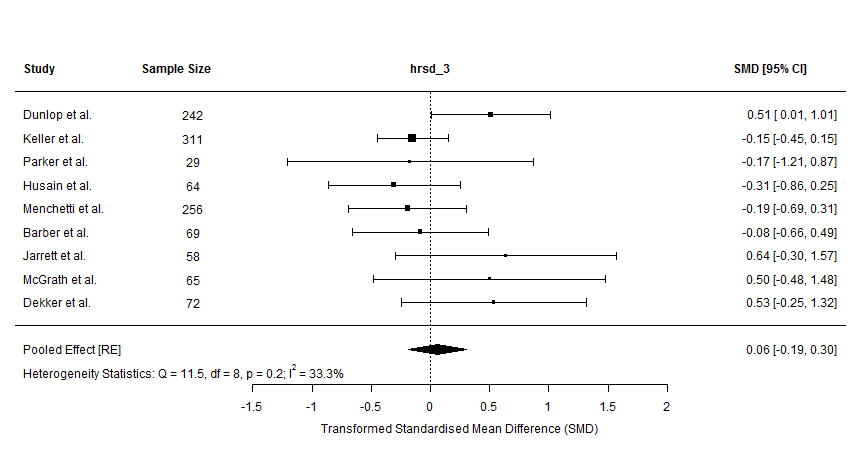 |
| 4 | 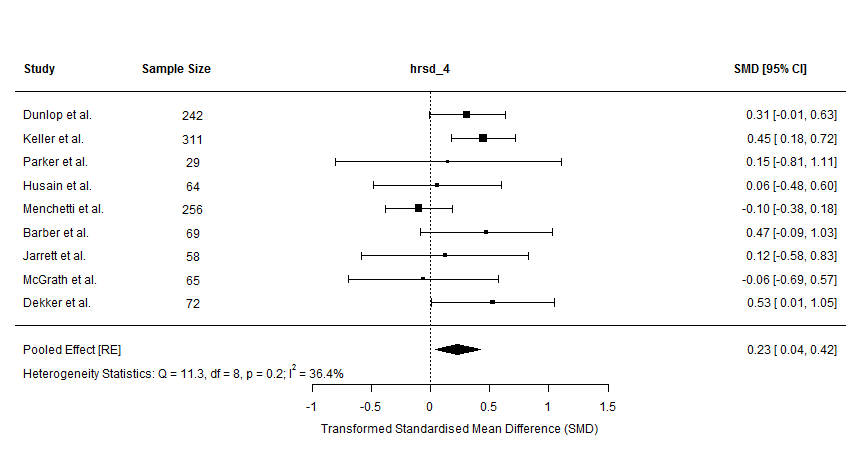 |
| 5 | 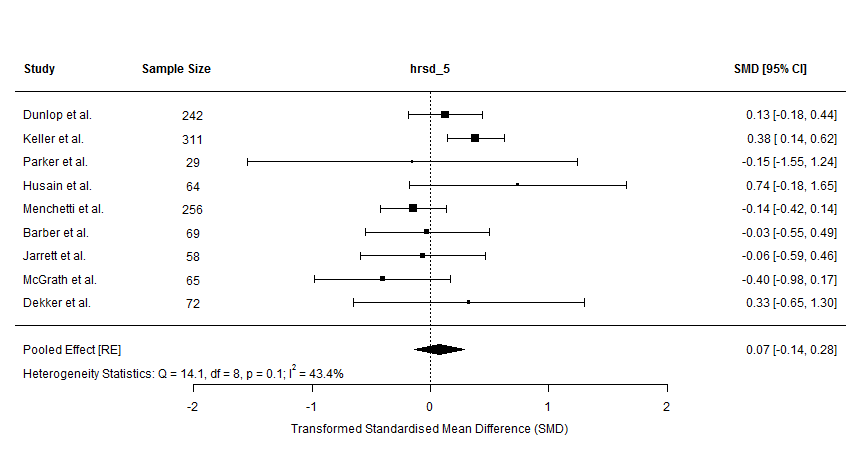 |
| 6 | 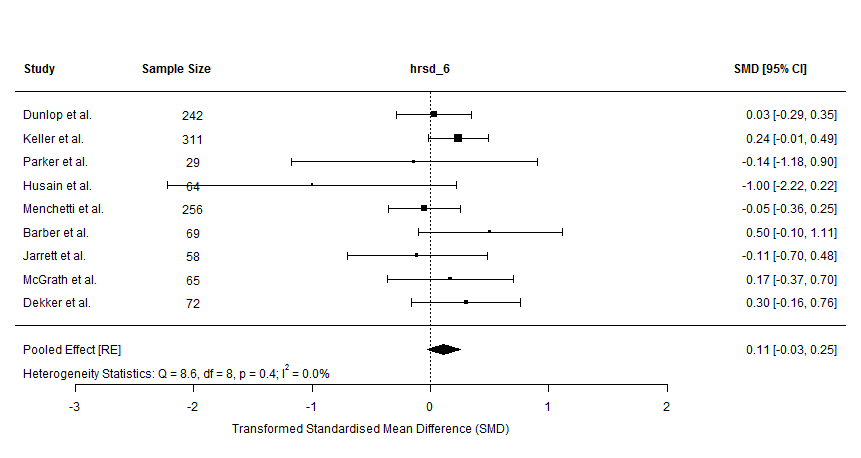 |
| 7 | 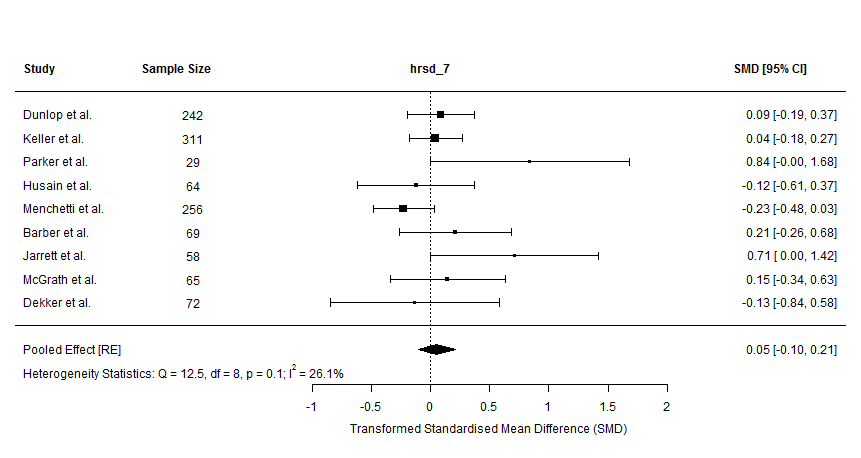 |
| 8 | 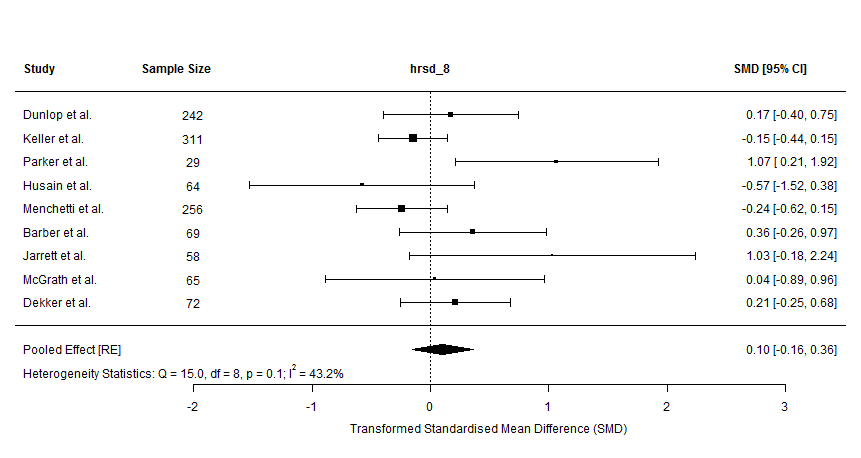 |
| 9 | 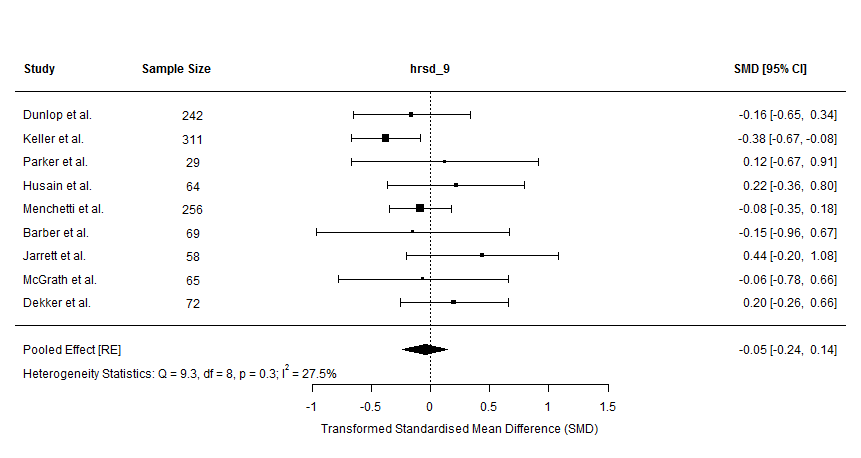 |
| 10 | 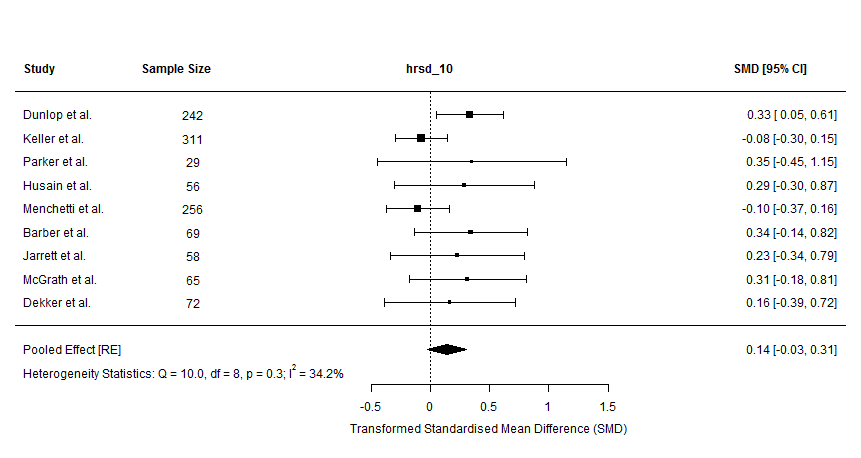 |
| 11 | 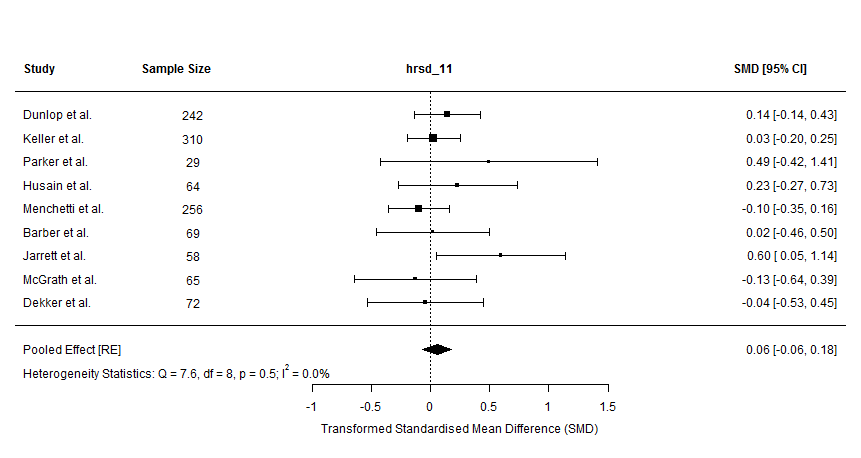 |
| 12 | 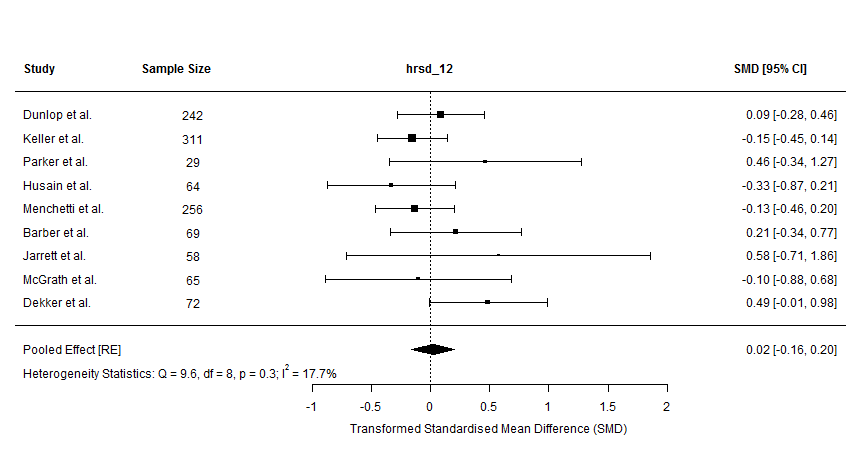 |
| 13 | 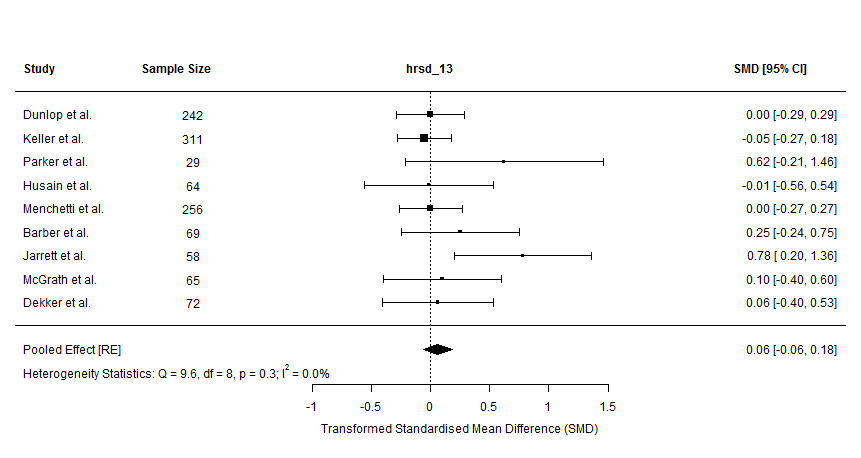 |
| 14 | 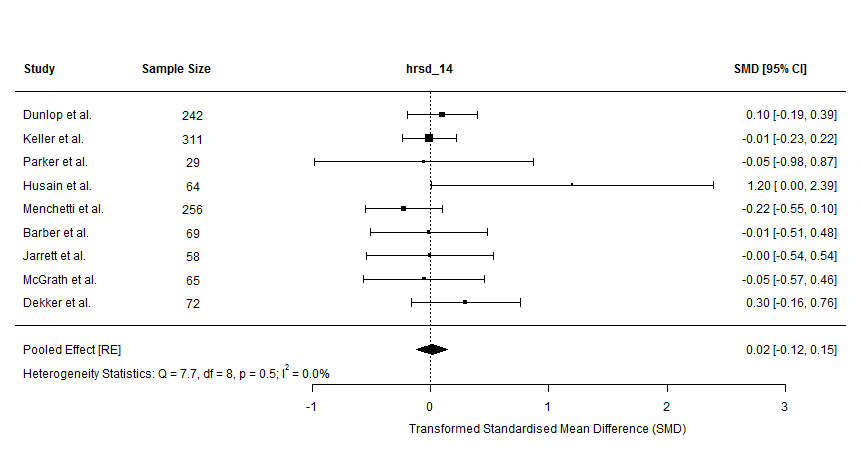 |
| 15 | 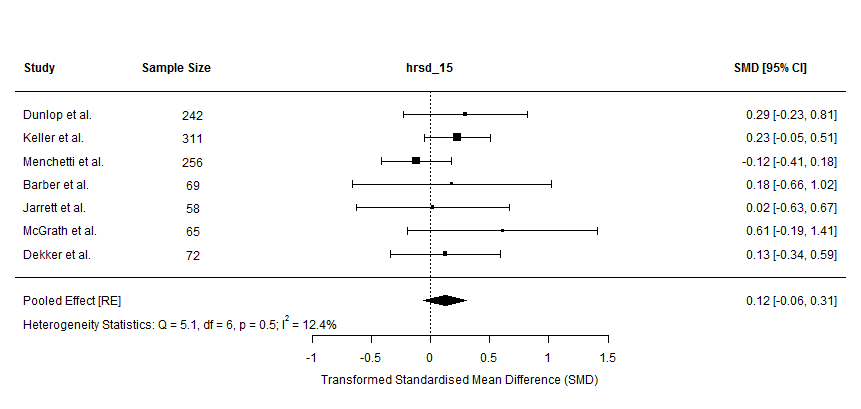 |
| 16 | 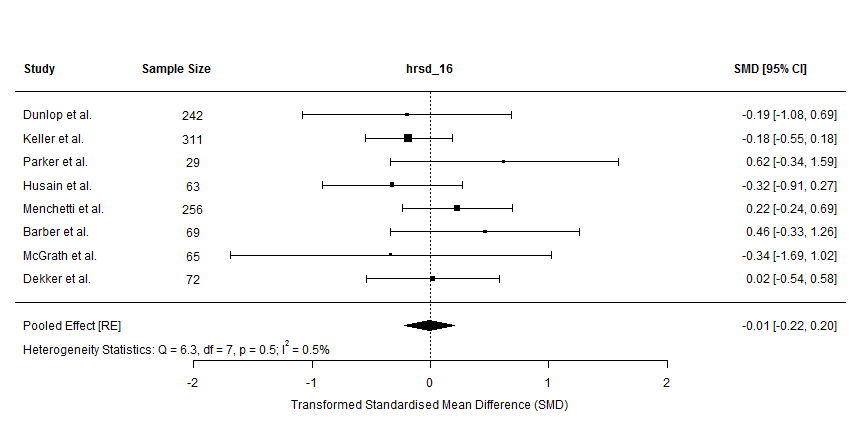 |
| 17 | 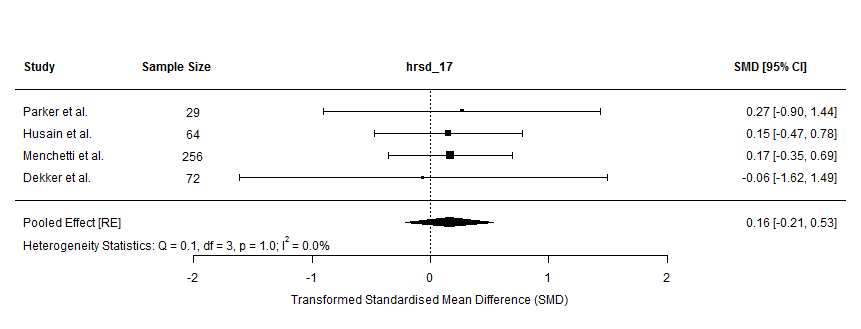 |
| 18 | 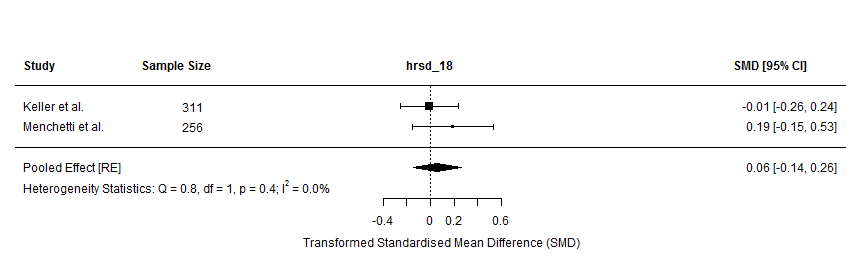 |
| 19 | 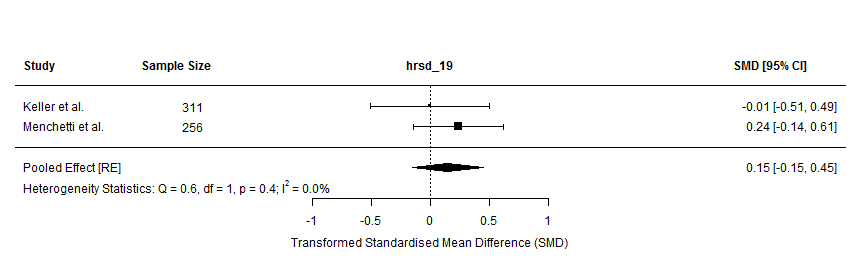 |
| 20 | 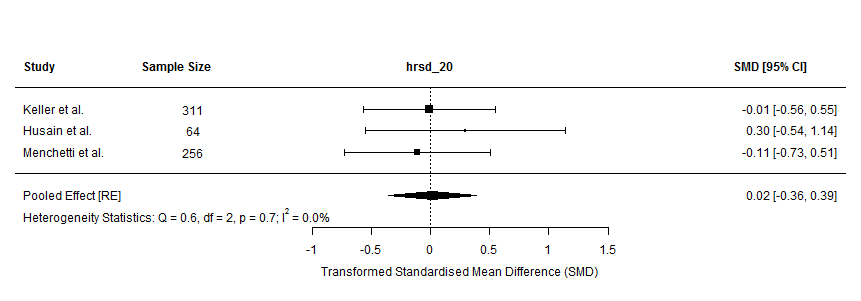 |
| 21 | 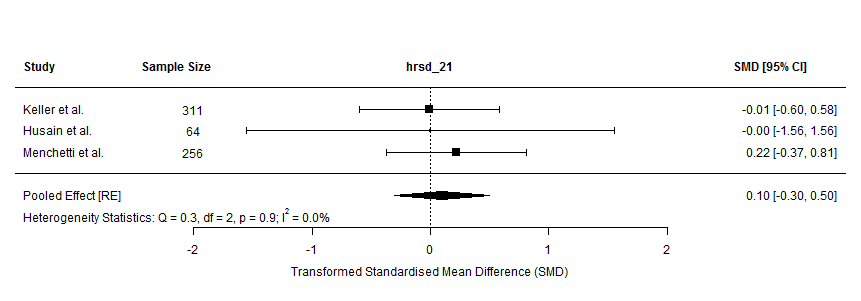 |

###

### Table S9: Individual symptom forest plots for the BDI

| BDI symptom | Forest Plot |
| --- | --- |
| 1 | 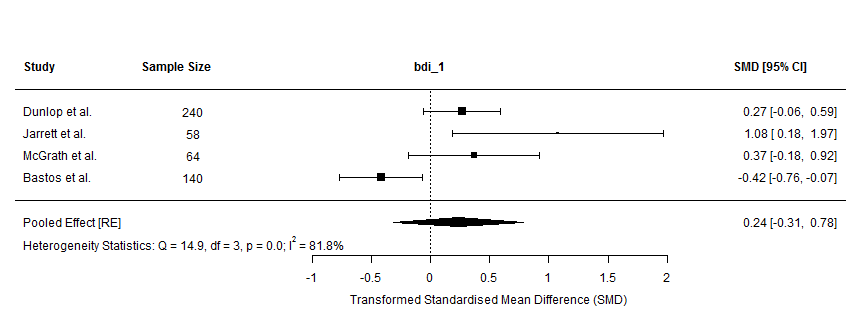 |
| 2 | 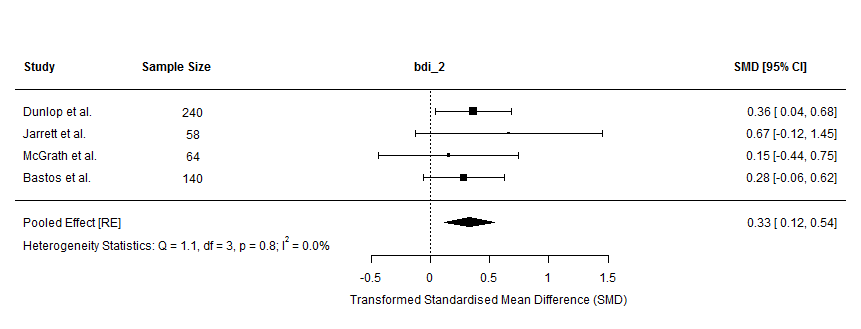 |
| 3 | 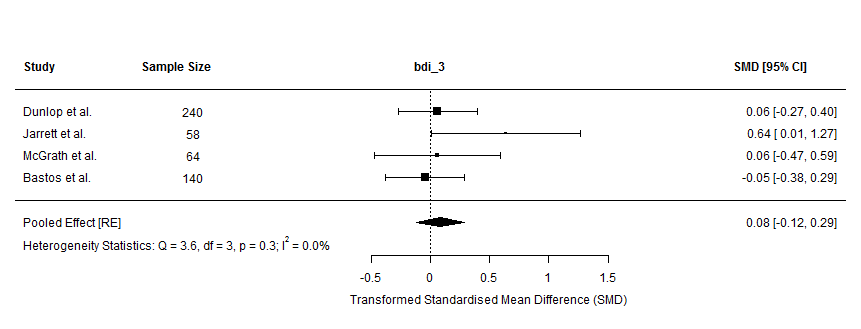 |
| 4 | 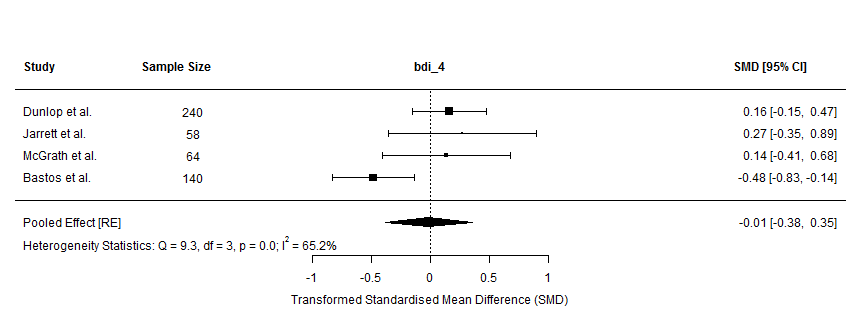 |
| 5 | 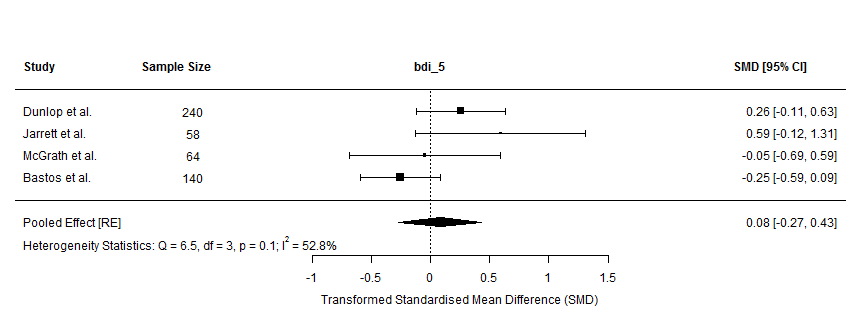 |
| 6 | 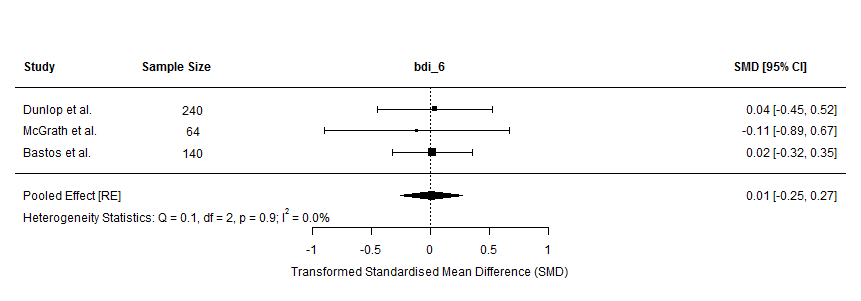 |
| 7 | 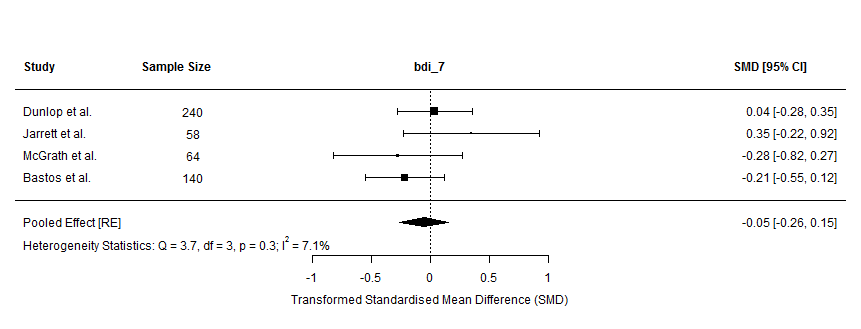 |
| 8 | 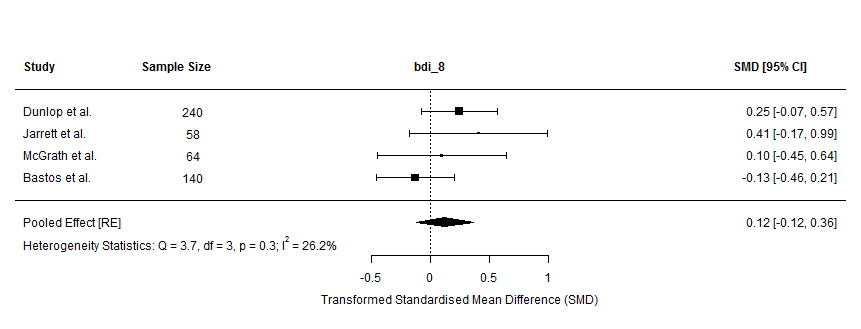 |
| 9 | 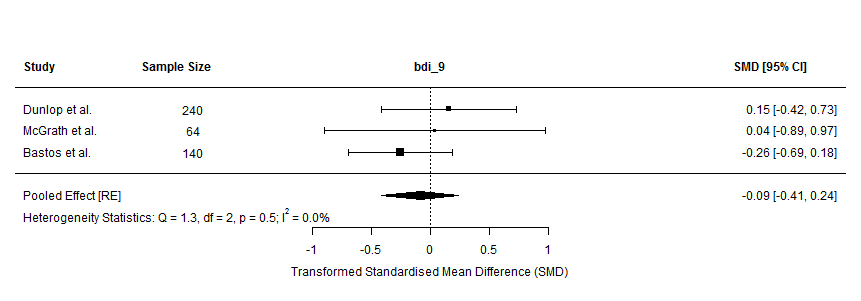 |
| 10 | 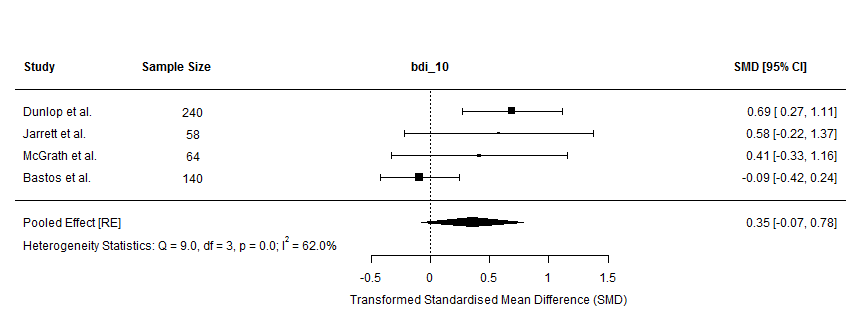 |
| 11 | 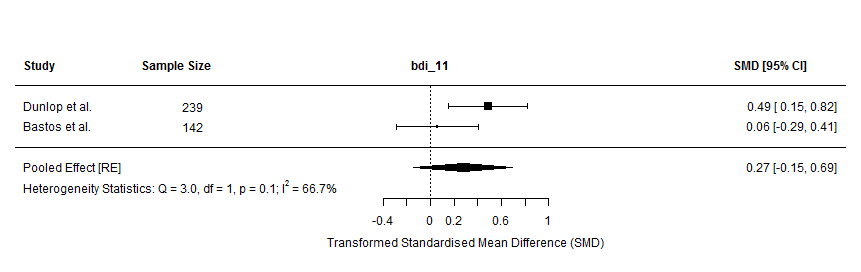 |
| 12 | 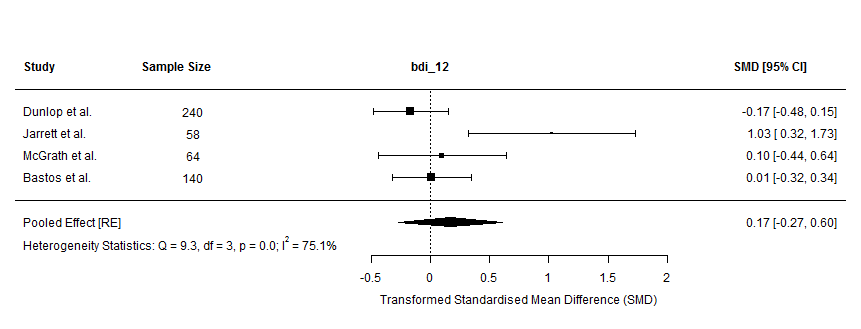 |
| 13 | 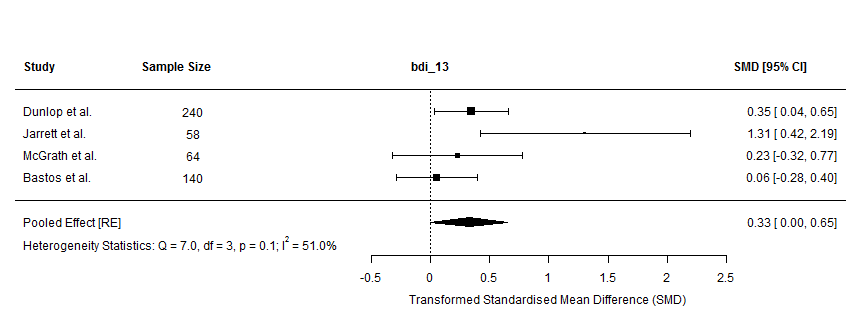 |
| 14 | 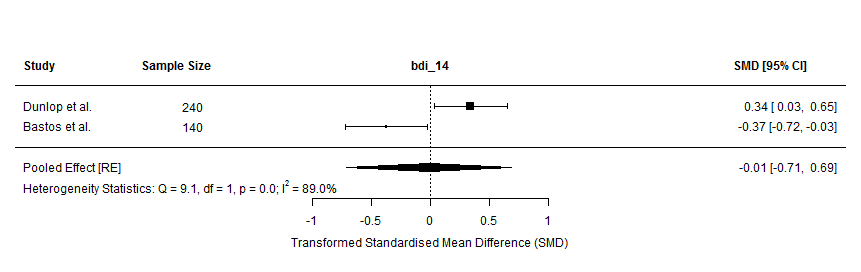 |
| 15 | 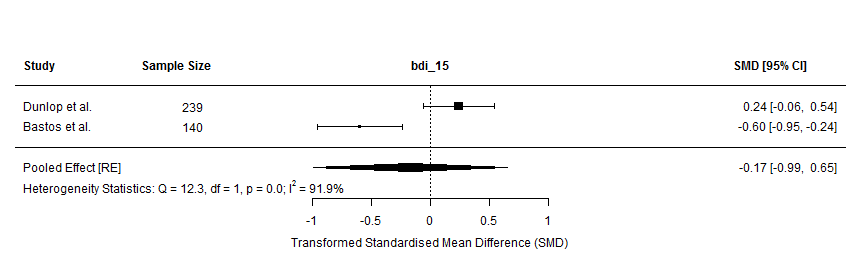 |
| 16 | 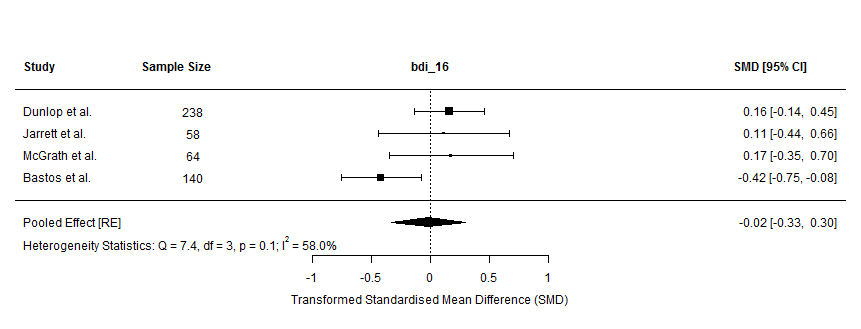 |
| 17 | 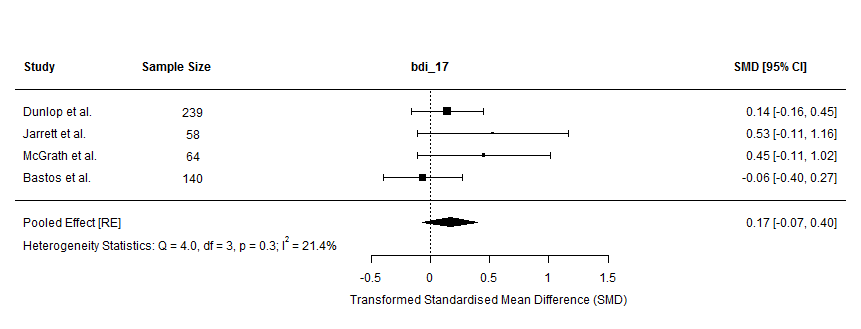 |
| 18 | 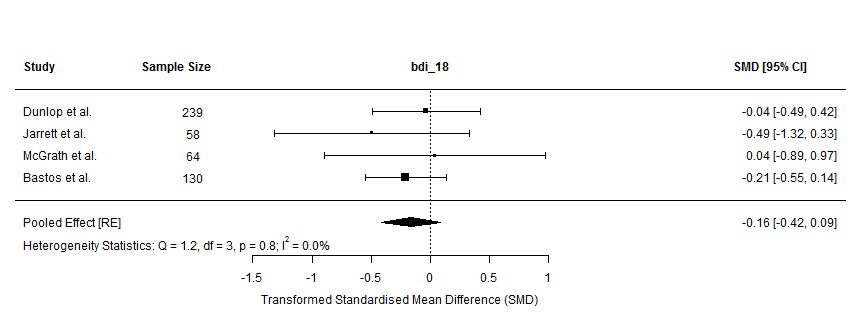 |
| 19 | 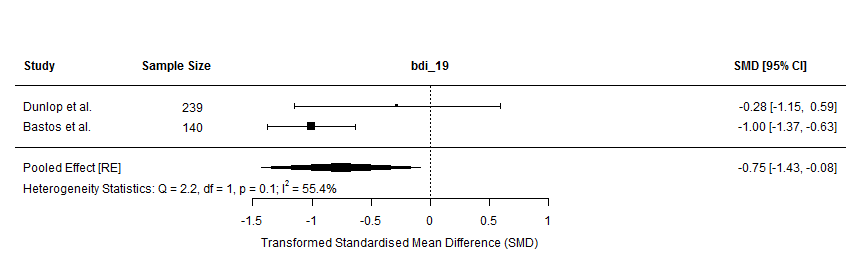 |
| 20 | 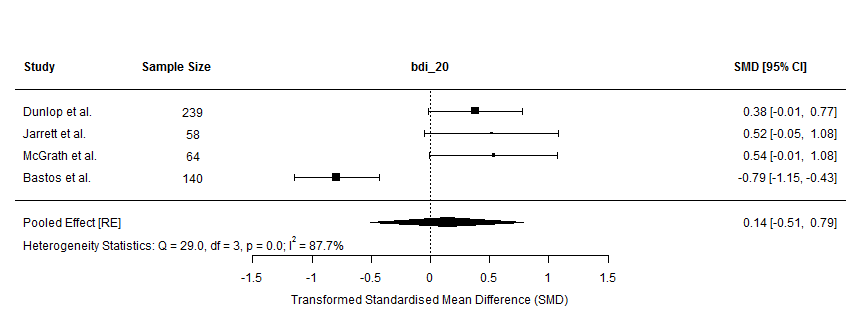 |
| 21 | 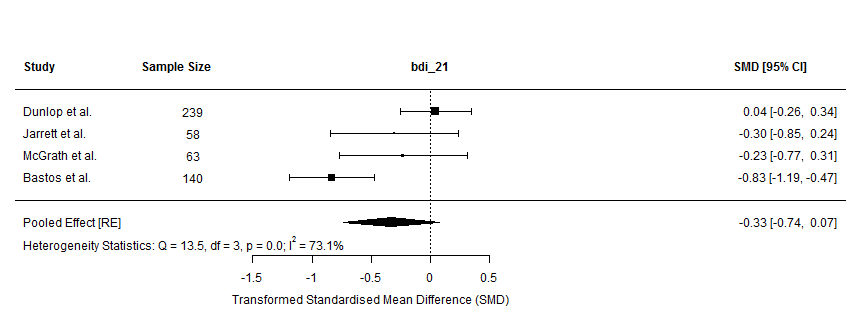 |

### Figure S6: Effect size comparison of HAM-D and BDI per symptom type


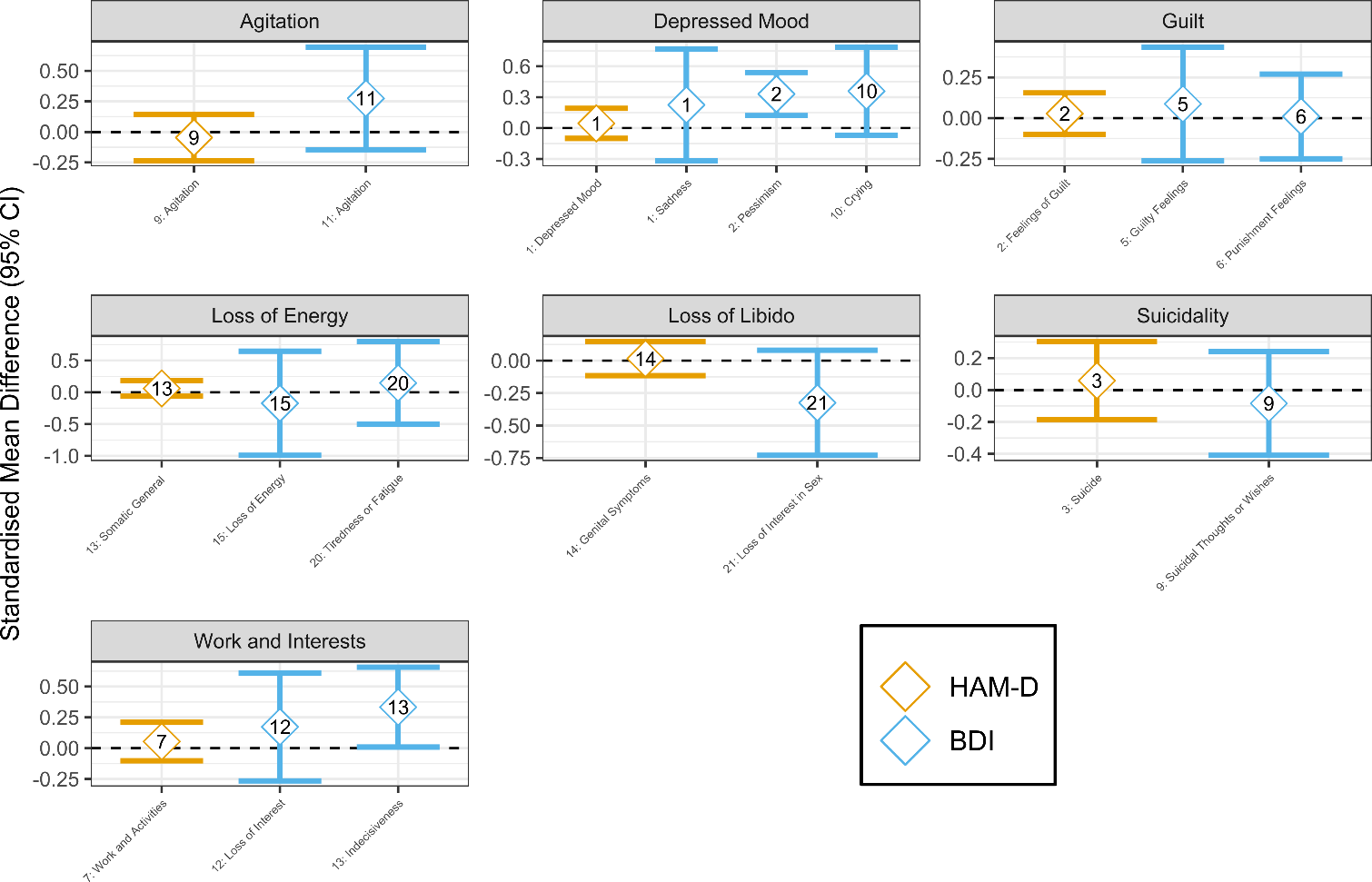


## Sensitivity Analyses

“Simple” ORs were computed following median split of each symptom item, setting items to 1 if a patient’s score was greater or equal to the median and scores lower than the median to 0 (if the median was 0, all scores above zero were set to 1).

Figure S7 shows pooled meta-analytic effect sizes for individual symptoms for each effect size metric used in meta-analyses. We also assessed effect size metrics intercorrelations for HAM-D and BDI scales, which were in medium-to-large range (Pearson’s r range: 0.69-0.97) with pORs and ORs being more similar to each other (Pearson’s r range: 0.93-0.97) than both OR-based metrics with SMDs (Pearson’s r range: 0.69-0.96). More detailed results of these sensitivity analyses are presented in Table S10 and Figure S8.

### Figure S7: Individual symptom effect sizes per effect size metric used in meta-analyses


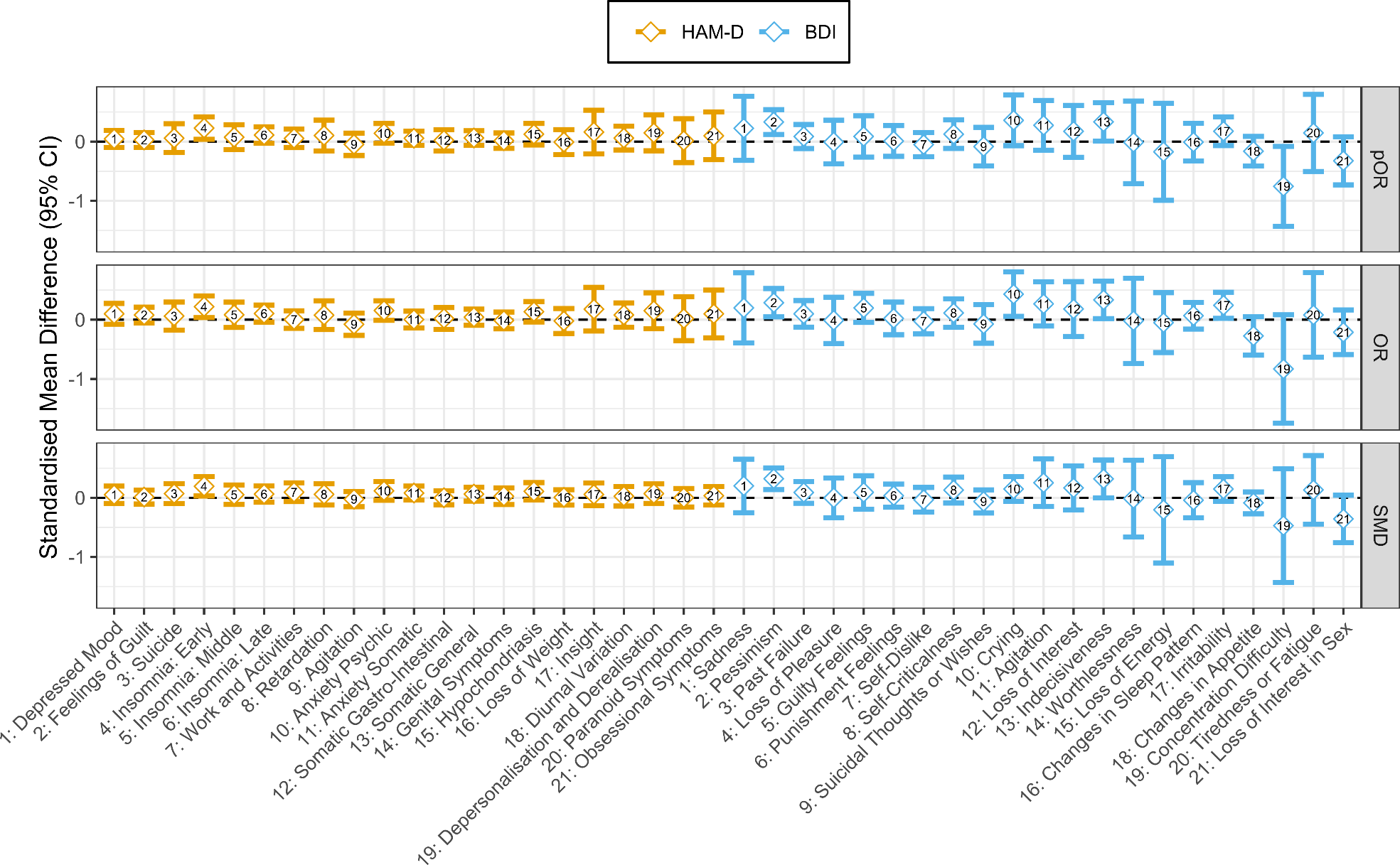


### Table S10: Correlations between meta-analytic effect sizes metrics

|  |  | HAM-D |  |  | BDI |  |
| --- | --- | --- | --- | --- | --- | --- |
|  | pOR | OR | SMD | pOR | OR | SMD |
| pOR | 1 |  |  | 1 |  |  |
| OR | 0.93 | 1 |  | 0.97 | 1 |  |
| SMD | 0.83 | 0.69 | 1 | 0.96 | 0.89 | 1 |

*Note*: HAM-D=Hamilton Rating Scale for Depression; BDI=Beck Depression Inventory; pOR=proportional Odds Ratio, OR=Odds Ratio; SMD=Standardised Mean Difference.

### Figure S8: Associations between meta-analytic effect size metrics


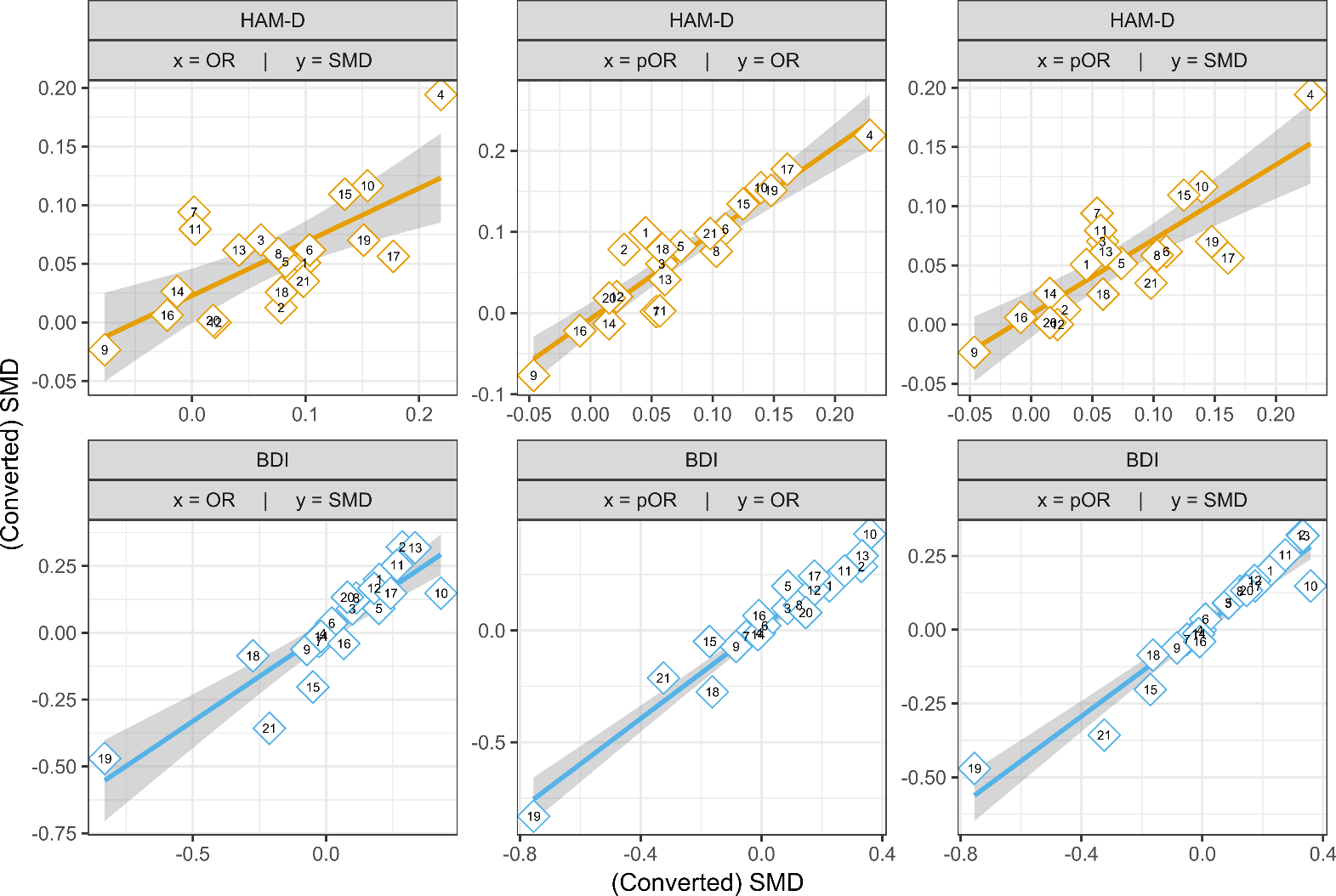


## Exploratory Comparison with Boschloo et al.

We performed two sets of exploratory comparisons. First, we correlated our individual HAM-D symptom effect sizes with theirs (cf. Table 2 of Boschloo et al.), which did not indicate an association between effect sizes (Pearson’s r=0.04, df=15, p=0.890; Fig. S9). Next, we repeated our individual symptom meta-analyses for the first 16 HAM-D items and restricted to those four RCTs that assessed CBT as psychotherapy of choice (cf. Additional file 6: Table S6). Although SMDs from these analyses are more closely related to results from Boschloo et al. (Pearson’s r=0.44, df=14, p=0.085; Fig. S10), the correlation is still of small-to-moderate size. A likely reason for this discrepancy is that we only meta-analysed four RCTs with a median of 394 patients per symptom, while meta-analysis of Boschloo et al. reported 1070 patients. This also highlights, however, that individual studies potentially introduce a lot of variability in symptom-specific comparative effectiveness.

### Figure S9: Effect size association to Boschloo et al.


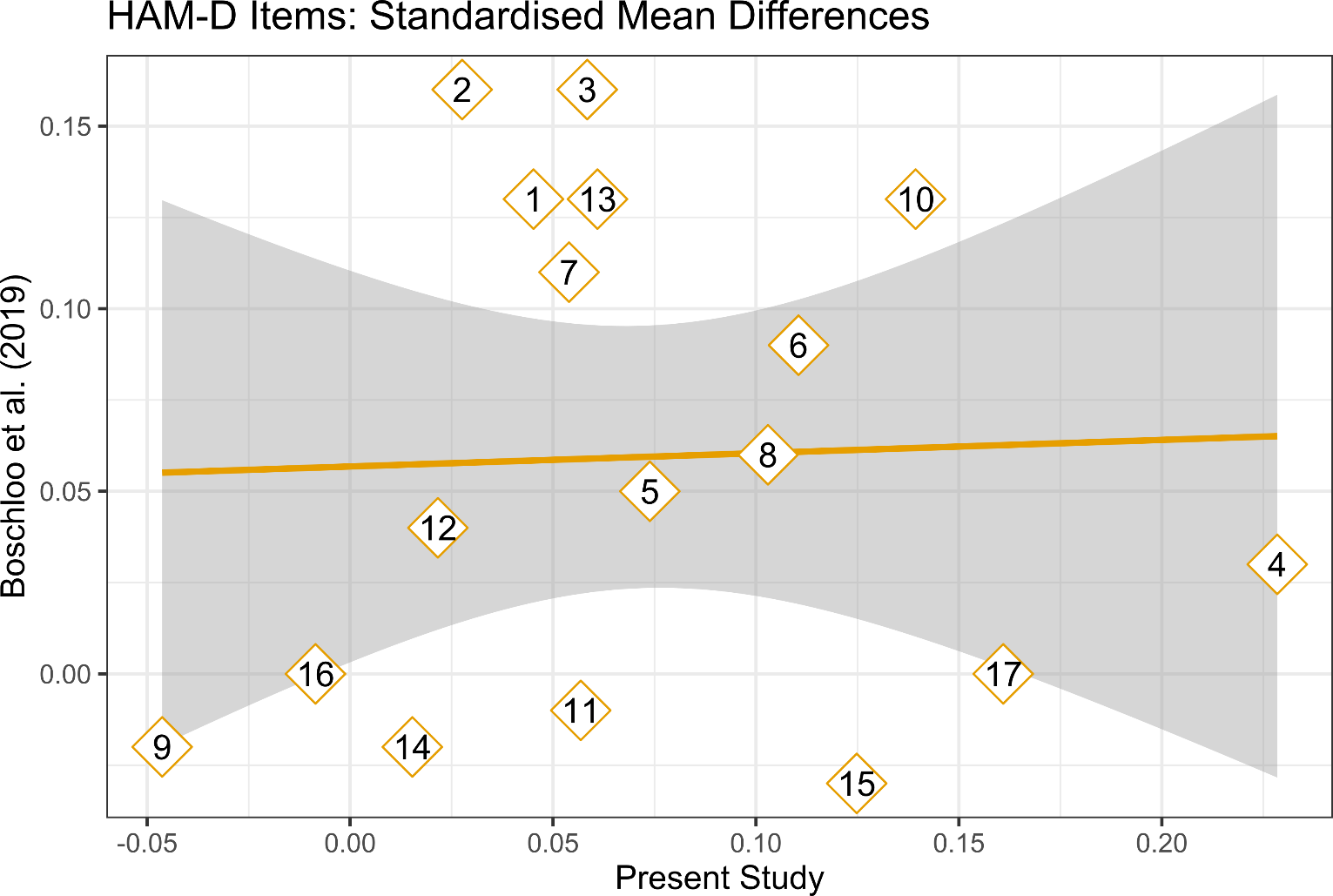


### Figure S10: Effect size association to Boschloo et al. for RCTs with CBT only


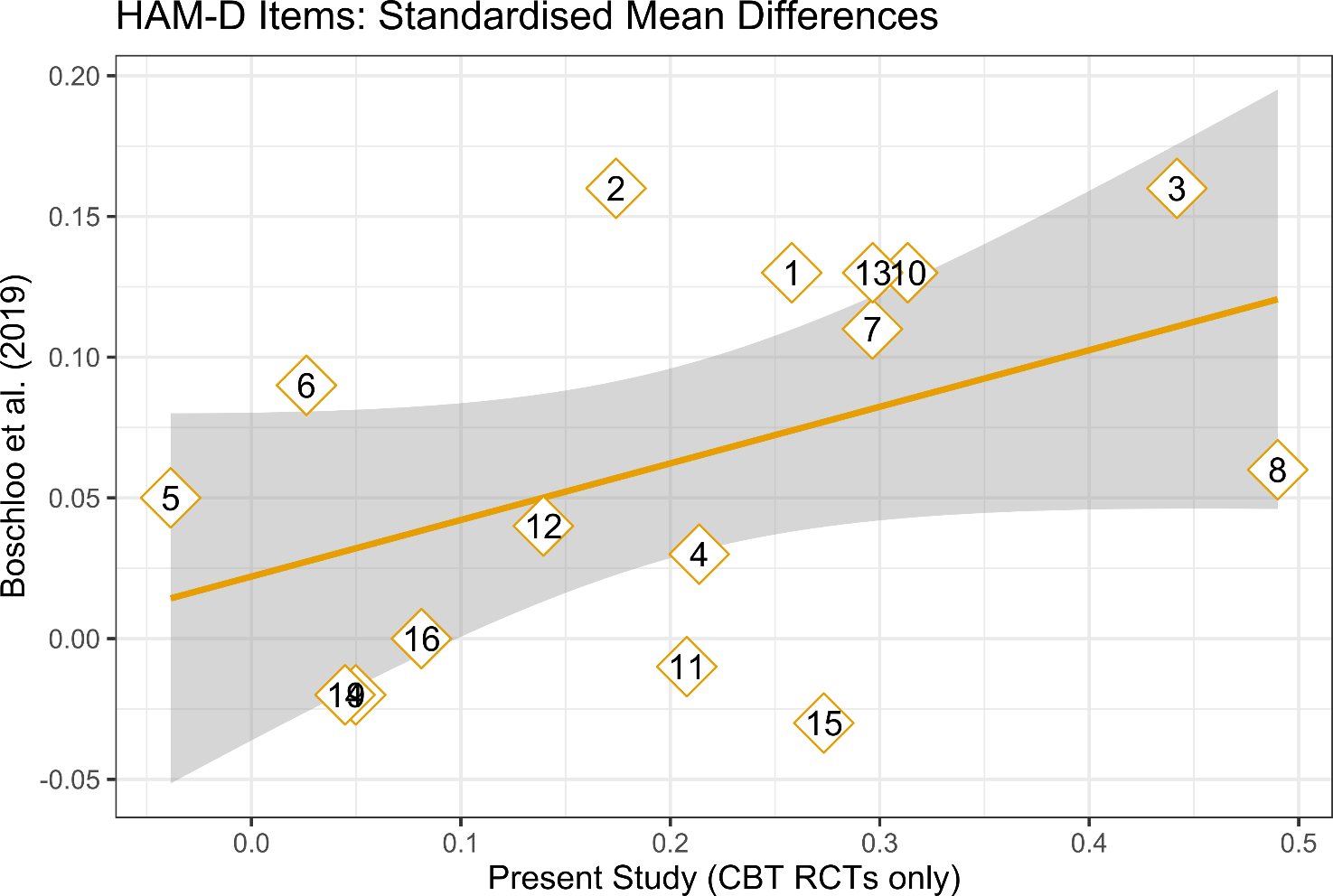

Supplement: Supplementary file 8 — Additional file 8: Tables S8-S10, Figs. S6-S10. Individual symptom meta-analysis results and related sensitivity and exploratory analyses. Table S8- Individual symptom forest plots for the HAM-D. Table S9- Individual symptom forest plots for the BDI. Table S10- Correlations between meta-analytic effect size metrics. Fig. S6- Effect size comparison of HAM-D and BDI per symptom type. Fig. S7- Individual symptom effect sizes per effect size metric used in meta-analyses. Fig. S8- Associations between meta-analytic effect size metrics. Fig. S9- Effect size association to Boschloo et al. Fig. S10- Effect size association to Boschloo et al. for RCTs with CBT only. [file 12916_2020_1623_MOESM8_ESM.docx]
