# Supplementary material for: Psychotherapy or medication for depression? Using individual symptom meta-analyses to derive a Symptom-Oriented Therapy (SOrT) metric for a personalised psychiatry
Source: BMC Med. 2020 Jun 5;18:170. doi: 10.1186/s12916-020-01623-9 (PMC7273646; doi:10.1186/s12916-020-01623-9)
Supplement: Supplementary file 9 — Additional file 9: Tables S11-S18, Fig. S11. Validation analyses of SOrT metric in MARS and PReDICT samples and related exploratory analyses. Table S11- Effect sizes of individual symptom meta-analyses with versus without Dunlop et al. Table S12- Linear regression analysis of 12-week HAM-D sum-scores on SOrT-based BDI treatment allocation match following valence split. Table S13- Linear regression analysis of 12-week HAM-D sum-scores on SOrT-based treatment allocation match following median split. Table S14- Linear regression analysis of 12-week HAM-D sum-scores on SOrT-based treatment allocation match following 2/3 extreme group split. Table S15- Treatment allocation match prediction using Boschloo et al.-based SOrT and sum-scores. Table S16- Regression-based treatment allocation match prediction using Boschloo et al.-based SOrT and sum-scores. Table S17- Association of symptom severity with updated SOrT scores. Table S18- Treatment allocation match prediction using updated SOrT metric. Fig. S11- Association and distributions of HAM-D and BDI SOrT scores in PReDICT. [file 12916_2020_1623_MOESM9_ESM.docx]

**Additional file 9**

## Step 2: Development and Validation of the Symptom-Oriented Therapy (SOrT) Metric

## Validation in PReDICT study

Table S11: Effect sizes of individual symptom meta-analyses with versus without Dunlop et al.

|  | HAM-D | | BDI | |
| --- | --- | --- | --- | --- |
| Item No | Dunlop et al. included | Dunlop et al. excluded | Dunlop et al. included | Dunlop et al. excluded |
| 1 | 0.04 | 0.05 | 0.25 | 0.24 |
| 2 | -0.01 | 0.03 | 0.31 | 0.33 |
| 3 | -0.07 | 0.06 | 0.15 | 0.08 |
| 4 | 0.21 | 0.23 | -0.07 | -0.01 |
| 5 | 0.06 | 0.07 | 0.03 | 0.08 |
| 6 | 0.13 | 0.11 | 0.00 | 0.01 |
| 7 | 0.07 | 0.05 | -0.08 | -0.05 |
| 8 | 0.11 | 0.10 | 0.06 | 0.12 |
| 9 | -0.02 | -0.05 | -0.20 | -0.09 |
| 10 | 0.08 | 0.14 | 0.19 | 0.35 |
| 11 | 0.04 | 0.06 | 0.06 | 0.27 |
| 12 | 0.02 | 0.02 | 0.33 | 0.17 |
| 13 | 0.10 | 0.06 | 0.44 | 0.33 |
| 14 | -0.01 | 0.02 | -0.37 | -0.01 |
| 15 | 0.11 | 0.12 | -0.60 | -0.17 |
| 16 | 0.01 | -0.01 | -0.08 | -0.02 |
| 17 | 0.16 | 0.16 | 0.25 | 0.17 |
| 18 |  |  | -0.22 | -0.16 |
| 19 |  |  | -1.00 | -0.75 |
| 20 |  |  | 0.07 | 0.14 |
| 21 |  |  | -0.49 | -0.33 |

### Figure S11: Association and distributions of HAM-D and BDI SOrT scores in PReDICT


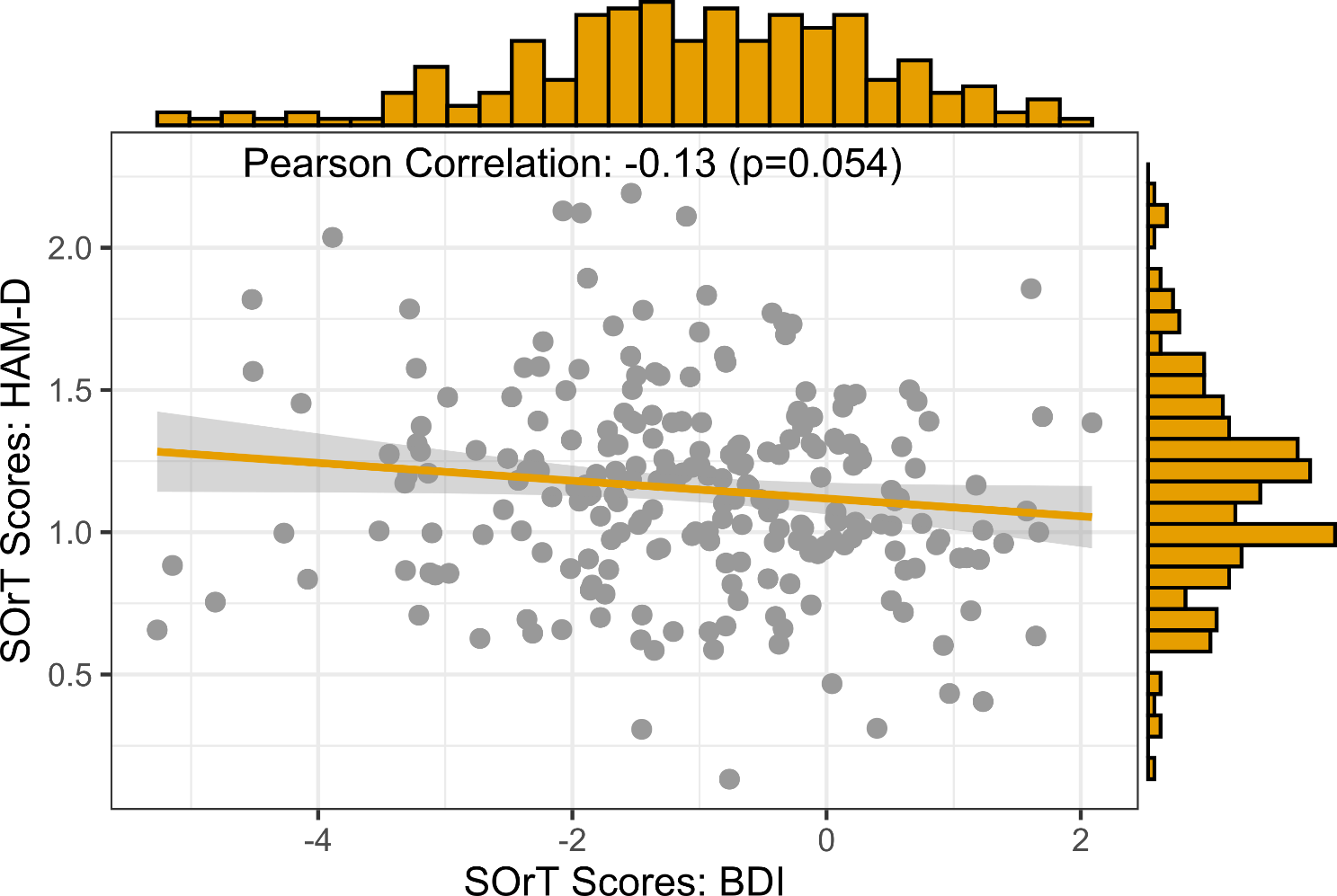


### Table S12: Linear regression analysis of 12-week HAM-D sum-scores on SOrT-based BDI treatment allocation match following valence split

|  | Unadjusted for baseline HAM-D |  | Adjusted for baseline HAM-D |  |
| --- | --- | --- | --- | --- |
| SOrT-Metric | Beta (SE) | P | Beta (SE) | P |
| Intercept | 8.03 (0.51) | <0.001 | -0.50 (2.27) | 0.824 |
| SOrT Match† | -0.42 (0.83) | 0.617 | -0.27 (0.81) | 0.738 |
| Baseline HAM-D Covariate | - | - | 0.44 (0.11) | <0.001 |

*Note*: n=237; † SOrT match is a binary variable (0=non-optimal, 1=optimal treatment allocation)

### Table S13: Linear regression analysis of 12-week HAM-D sum-scores on SOrT-based treatment allocation match following median split

|  | Unadjusted for baseline HAM-D |  | Adjusted for baseline HAM-D |  |
| --- | --- | --- | --- | --- |
| SOrT-Metric | Beta (SE) | P | Beta (SE) | P |
| HAM-D |  |  |  |  |
| Intercept | 7.17 (0.56) | <0.001 | -0.78 (2.17) | 0.719 |
| SOrT Match† | 1.34 (0.79) | 0.091 | 0.76 (0.78) | 0.330 |
| Baseline HAM-D Covariate | - | - | 0.43 (0.11) | <0.001 |
| BDI |  |  |  |  |
| Intercept | 8.09 (0.55) | <0.001 | -0.42 (2.26) | 0.812 |
| SOrT Match† | -0.47 (0.81) | 0.563 | -0.45 (0.78) | 0.566 |
| Baseline HAM-D Covariate | - | - | 0.44 (0.11) | <0.001 |

*Note*: HAM-D: n=242; BDI: n=235; † SOrT match is a binary variable (0=non-optimal, 1=optimal treatment allocation)

### Table S14: Linear regression analysis of 12-week HAM-D sum-scores on SOrT-based treatment allocation match following 2/3 extreme group split

|  | Unadjusted for baseline HAM-D |  | Adjusted for baseline HAM-D |  |
| --- | --- | --- | --- | --- |
| SOrT-Metric | Beta (SE) | P | Beta (SE) | P |
| HAM-D |  |  |  |  |
| Intercept | 7.08 (0.69) | <0.001 | -0.87 (2.62) | 0.740 |
| SOrT Match† | 1.77 (0.99) | 0.074 | 0.95 (1.00) | 0.343 |
| Baseline HAM-D Covariate | - | - | 0.43 (0.14) | 0.002 |
| BDI |  |  |  |  |
| Intercept | 7.66 (0.72) | <0.001 | -0.34 (2.76) | 0.903 |
| SOrT Match† | -0.65 (0.98) | 0.510 | -0.72 (0.96) | 0.453 |
| Baseline HAM-D Covariate | - | - | 0.42 (0.14) | 0.003 |

*Note*: HAM-D: n=161; BDI: n=157; † SOrT match is a binary variable (0=non-optimal, 1=optimal treatment allocation)

## Exploratory Analyses

###

We conducted two further sets of exploratory analyses to compare our results with those by Boschloo *et al.* [97]. In particular, we (i) created another SOrT allocation metric for the HAM-D and (ii) created and tested their sum-score based allocation metric of symptoms differentially affected by ADM and psychotherapy in their study (depressed mood, feelings of guilt, suicidal thoughts, psychic anxiety, & general somatic symptoms). Testing optimal versus non-optimal treatment match by median split and extreme group comparison (as before) did not reveal significantly lower HAM-D scores at end of treatment for the Boschloo et al.-based SOrT metric or for the Boschloo et al.-based sum-score metric (Tables S15-S16).

Because the SOrT metric showed unexpected associations with depressive symptom severity, we also deemed it important to adjust computation of the metric in a way that prevents this potential artifact and optimises the metric on its treatment differentiation aspect. Accordingly, we adjusted the formula of the metric to $SOrT=\frac{\sum_{i} {m_{i}s}_{i}}{\sum_{i} s_{i}}$, so that we divided the initial terms of the formula by the symptom sum-score (as a reminder: *m* equals the meta-analytic effect size [favouring ADM or psychotherapy] as [converted] SMD, *s* equals the symptom score on BDI or HAM-D, and *i* equals a specific symptom item). Applying this updated formula removed correlations with symptom severity (Table S17). When evaluating (updated) SOrT-based treatment allocation into optimal versus non-optimal treatment arms in the PReDICT sample, we found evidence contrary to expectation for the HAM-D SOrT metric in that patients in non-optimal treatment had lower depressive symptoms at study endpoint. For the BDI SOrT metric, there was some evidence (median-split & extreme groups, but not valence) for significantly lower depressive symptoms for those patients who were allocated to their optimal treatment (Table S18).

Exploratory analyses showed that Boschloo et al.-based allocation metrics did not improve treatment allocation decisions. For the updated SOrT metric, there appeared some benefit for BDI-based SOrT allocation and clear disadvantages for HAM-D-based SOrT allocation. The significant disadvantage of HAM-D-based SOrT allocation is surprising and counterintuitive as it would mean patients exhibiting symptoms that are better treated with ADM or psychotherapy (as shown by meta-analysis) actually fared worse when receiving this treatment. It may thus be likely that this counterintuitive finding has arisen by chance, but conclusive statements will only be possible following replication. Independent from exact interpretations, however, results suggest that symptom-based metrics do not offer reliable advantages for allocation to psychotherapy versus ADM.

### Table S15: Treatment allocation match prediction using Boschloo et al.-based SOrT and sum-scores

|  |  | 12-Week HAM-D, Mean (SD) | |  |
| --- | --- | --- | --- | --- |
| Method | n | Optimal | Non-Optimal | P-value^†^ |
| Boschloo et al.-based SOrT score | | | | |
| Median-split | 242 | 8.17 (6.59) | 7.52 (5.74) | 0.414 |
| Extreme groups | 160 | 7.98 (6.54) | 7.82 (5.67) | 0.875 |
| Boschloo et al.-based sum-score | | | | |
| Median-split | 242 | 8.47 (6.45) | 7.44 (6.45) | 0.204 |
| Extreme groups | 137 | 8.75 (6.73) | 7.77 (6.33) | 0.380 |

*Note*: ^†^P-values are based on independent-samples t-tests.

### Table S16: Regression-based treatment allocation match prediction using Boschloo et al.-based SOrT and sum-scores

|  | Unadjusted for baseline HAM-D | | Adjusted for baseline HAM-D | |
| --- | --- | --- | --- | --- |
| SOrT-Metric | Beta (SE) | P | Beta (SE) | P |
| Boschloo et al.-based SOrT score | | | | |
| Intercept | 7.52 (0.55) | <0.001 | -0.89 (2.19) | 0.685 |
| Match† | 0.65 (0.79) | 0.414 | -0.25 (0.80) | 0.754 |
| Baseline HAM-D Covariate | - | - | 0.46 (0.12) | <0.001 |
| Boschloo et al.-based sum-score | | | | |
| Intercept | 7.44 (0.50) | <0.001 | -0.85 (2.17) | 0.696 |
| Match† | 1.04 (0.81) | 0.203 | 0.66 (0.80) | 0.408 |
| Baseline HAM-D Covariate | - | - | 0.44 (0.11) | <0.001 |

*Note*: Sample size: n=242. † Match is a binary variable (0=non-optimal, 1=optimal treatment allocation)

### Table S17: Association of symptom severity with updated SOrT scores

|  | MARS | | PReDICT | |
| --- | --- | --- | --- | --- |
|  | Pearson’s r | P | Pearson’s r | P |
| HAM-D SOrT^†^ | 0.07 | 0.165 | 0.01 | 0.933 |
| BDI SOrT^†^ | -0.03 | 0.489 | -0.05 | 0.408 |

*Note*: ^†^The SOrT metric is calculated based on an updated formula adjusting for the median meta-analytic effect size and correlations are computed to baseline symptom severity on respective scale.

### Table S18: Treatment allocation match prediction using updated SOrT metric

|  |  | 12-Week HAM-D, Mean (SD) | |  |
| --- | --- | --- | --- | --- |
| Method | n | Optimal | Non-Optimal | P-value^†^ |
| HAM-D-based SOrT allocation | |  |  |  |
| Valence |  | - | - | - |
| Median-split | 242 | 9.34 (5.84) | 6.38 (5.84) | <0.001 |
| Extreme groups | 162 | 10.19 (6.99) | 5.94 (6.69) | <0.001 |
| BDI-based SOrT allocation | |  |  |  |
| Valence | 242 | 8.10 (6.11) | 7.64 (6.26) | 0.582 |
| Median-split | 242 | 6.65 (6.13) | 9.08 (6.04) | 0.003 |
| Extreme groups | 145 | 6.21 (6.89) | 9.64 (7.04) | 0.020 |

*Note*: ^†^P-values are based on independent-samples t-tests.
