# Supplementary material for: Psychotherapy or medication for depression? Using individual symptom meta-analyses to derive a Symptom-Oriented Therapy (SOrT) metric for a personalised psychiatry
Source: BMC Med. 2020 Jun 5;18:170. doi: 10.1186/s12916-020-01623-9 (PMC7273646; doi:10.1186/s12916-020-01623-9)
Supplement: Supplementary file 10 — Additional file 10. Discussion on nominally significant treatment differences of psychotherapy and ADM for specific depressive symptoms. [file 12916_2020_1623_MOESM10_ESM.docx]

**Additional file 10**

# Discussion

## Nominally significant treatment differences of psychotherapy and ADM for specific depressive symptoms

Symptom-specific meta-analyses showed nominally significant differential treatment effects of psychotherapy or ADM for specific depressive symptoms. For the HAM-D, the *Insomnia: Early* symptom (relating to difficulty falling asleep) was more favourably treated by ADM compared to psychotherapy. There were, however, no differences for other insomnia items relating to disturbed sleep during the night and early awakenings. Similar symptom-specific meta-analytic work on the HAM-D by Boschloo and colleagues [96], to which we compared our results on an exploratory basis, did not show differences in *Insomnia: Early*, nor did studies looking at symptom clusters and residual symptoms over follow-up [26, 38]. For the BDI, we found ADM to be favourable to psychotherapy for reducing *pessimism* and *indecisiveness* while *concentration difficulties* were better addressed by psychotherapy. Although our analyses are the first to evaluate symptom-specific effects on the BDI, these findings do not align with prior studies in terms of symptom content [26, 38, 96]. Based on these discrepancies to prior literature, it remains unclear whether true symptom-specific treatment differences between psychotherapy and ADM exist or whether reports from our study and previous literature reflect false positive findings.
