# Supplementary material for: Psychotherapy or medication for depression? Using individual symptom meta-analyses to derive a Symptom-Oriented Therapy (SOrT) metric for a personalised psychiatry
Source: BMC Med. 2020 Jun 5;18:170. doi: 10.1186/s12916-020-01623-9 (PMC7273646; doi:10.1186/s12916-020-01623-9)
Supplement: Supplementary file 11 — Additional file 11: Tables S19-S20. Overview of available data and materials from original studies and presented work. Table S19 Availability of Original Study Data. Table S20. Online File Overview as Available on https://osf.io/qzjc9/. [file 12916_2020_1623_MOESM11_ESM.docx]

**Additional File 11**

## Availability of data and materials

### Table S19: Availability of Data and Materials

| Study | Success/ failure in contacting study authors | Any data obtained from study (yes /no) | Type of data obtained^1^ | Permission to share data |
| --- | --- | --- | --- | --- |
| Barber *et al.* [1] | Success | Yes | TL-I | A reasonable request for data can be made to original authors |
| Bastos *et al.* [2] | Success | Yes | TL-I | A reasonable request for data can be made to original authors |
| Bedi *et al.* [3] | Failure | - | - | - |
| Blackburn *et al.* [4] | Failure | - | - | - |
| Blackburn *et al.* [5] | Failure | - | - | - |
| Blom *et al.* [6] | Failure | - | - | - |
| David *et al.* [7] | Failure | - | - | - |
| Dekker *et al.* [8] | Success | Yes | IL | A reasonable request for data can be made to original authors |
| DeRubeis *et al.* [9] | Failure | - | - | - |
| DiMascio *et al.* [10] | Success | No | - | - |
| Dimidjian *et al.* [11] | Failure | - | - | - |
| Dunlop *et al.* [12] | Success | Yes | IL | A reasonable request for data can be made to original authors |
| Elkin *et al.* [13] | Failure | - | - | - |
| Frank *et al.* [14] | Success | No | - | - |
| Harkness *et al.* [15] | Success | No | - | - |
| Hollon *et al.* [16] | Success |  |  |  |
| Husain *et al.* [17] | Success | Yes | TL-I | A reasonable request for data can be made to original authors |
| Jarrett *et al.* [18] | Success | Yes | TL-I | A reasonable request for data can be made to original authors |
| Keller *et al.* [19] | Success | Yes | TL-I | A reasonable request for data can be made to original authors |
| Kennedy *et al.* [20] | Failure | - | - | - |
| Lopez Rodriguez *et al.* [21] | Success | No | - | - |
| Martin *et al.* [22] | Failure | - | - | - |
| McGrath *et al.* [23] | Success | Yes | IL | A reasonable request for data can be made to original authors |
| McKnight *et al.* [24] | Failure | - | - | - |
| McLean *et al.* [25] | Failure | - | - | - |
| Menchetti *et al.* [26] | Success | Yes | TL-I | A reasonable request for data can be made to original authors |
| Miranda *et al.* [27] | Failure | - | - | - |
| Moradveisi *et al.* [28] | Success | Yes | TL-S | A reasonable request for data can be made to original authors |
| Murphy *et al.* [29] | Failure | - | - | - |
| Mynors-Wallis *et al.* [30] | Failure | - | - | - |
| Mynors-Wallis *et al.* [31] | Failure | - | - | - |
| Parker *et al.* [32] | Success | Yes | IL |  |
| Rush *et al.* [33] | Failure | - | - | - |
| Salminen *et al.* [34] | Failure | - | - | - |
| Scott & Freeman [35] | Failure | - | - | - |
| Shamsaei *et al.* [36] | Success |  |  |  |
| Thompson *et al.* [37] | Success | No | - | - |
| Zu *et al.* [38] | Failure | - | - | - |

Note: ^1^The type of data obtained can be either: IL= raw data on individual level requiring our own computation of effect sizes for individual symptoms, TL-I=individual symptom data on treatment level (allowing no identification of individual patients), or TL-S=sum-score data on treatment level (prohibiting inclusion of meta-analysis on individual symptom level)

###

### Table S20: Online File Overview as Available on <https://osf.io/qzjc9/>

| File | Content |
| --- | --- |
| Search_Results.xlsx | Information on literature search and associated study inclusions and exclusions |
| DataExtractionSheet.xlsx | Data extracted from original RCTs |
| RiskOfBias.xlsx | Agreed upon Risk of Bias ratings with sheet 1 highlighting final ratings and sheet 2 describing reasons for ratings |
| Scripts |  |
| functions.R | R functions programmed to facilitate various project steps (e.g., effect size conversion, meta-analysis loop, etc.) |
| RoB.R | Visualisation of Risk of Bias ratings |
| StudyPreprocessing.R | Details on how individual study data were processed |
| StudyAggregation.R | Details how individual studies were aggregated in preparation for individual symptom meta-analyses |
| StudyAggregation_OR.R | Identical to StudyAggregation.R but used to aggregate data for OR sensitivity meta-analyses |
| StudyAggregation_SMD.R | Identical to StudyAggregation.R but used to aggregate data for SMD sensitivity meta-analyses |
| SumScore_MetaAnalyses.R | Sum-score meta-analyses based on data extracted from original study manuscripts |
| SOrT_MetaAnalyses.R | Individual symptom meta-analyses |
| SOrT_MetaAnalyses_Sensitivity.R | Sensitivity analyses of individual symptom meta-analyses |
| SOrT_Validation_MARS | Validation analyses in MARS sample |
| SOrT_Validation_PReDICT | Validation analyses in PReDICT sample |
| Data |  |
| RoB.csv | Risk of Bias rating data |
| SumScoreData.csv | Data for sum-score meta-analyses |
| MetaAnalysisData.RData | Main individual symptom meta-analysis effect sizes |
| MetaAnalysisData_OR.RData | Sensitivity individual symptom meta-analysis OR effect sizes |
| MetaAnalysisData_SMD.RData | Sensitivity individual symptom meta-analysis SMD effect sizes |
| MetaAnalysisResults.RData | Main individual symptom meta-analysis results |
| MetaAnalysisData_OR.RData | Sensitivity individual symptom meta-analysis results for OR effect size |
| MetaAnalysisData_SMD.RData | Sensitivity individual symptom meta-analysis results for SMD effect size |
